# High-performance bifunctional porous non-noble metal phosphide catalyst for overall water splitting

**DOI:** 10.1038/s41467-018-04746-z

**Published:** 2018-06-29

**Authors:** Fang Yu, Haiqing Zhou, Yufeng Huang, Jingying Sun, Fan Qin, Jiming Bao, William A. Goddard, Shuo Chen, Zhifeng Ren

**Affiliations:** 1 Department of Physics and TcSUH, University of Houston, Houston, TX 77204 USA; 2 Key Laboratory of Low-Dimensional Quantum Structures and Quantum Control of Ministry of Education, School of Physics and Electronics, Hunan Normal University, Changsha, 410081 China; 3 Materials and Process Simulation Center (139-74), California Institute of Technology, Pasadena, CA 91125 USA; 4 Department of Electrical and Computer Engineering, University of Houston, Houston, TX 77204 USA

## Abstract

Water electrolysis is an advanced energy conversion technology to produce hydrogen as a clean and sustainable chemical fuel, which potentially stores the abundant but intermittent renewable energy sources scalably. Since the overall water splitting is an uphill reaction in low efficiency, innovative breakthroughs are desirable to greatly improve the efficiency by rationally designing non-precious metal-based robust bifunctional catalysts for promoting both the cathodic hydrogen evolution and anodic oxygen evolution reactions. We report a hybrid catalyst constructed by iron and dinickel phosphides on nickel foams that drives both the hydrogen and oxygen evolution reactions well in base, and thus substantially expedites overall water splitting at 10 mA cm^−2^ with 1.42 V, which outperforms the integrated iridium (IV) oxide and platinum couple (1.57 V), and are among the best activities currently. Especially, it delivers 500 mA cm^−2^ at 1.72 V without decay even after the durability test for 40 h, providing great potential for large-scale applications.

## Abstract (web summary)

Water electrolysis provides a carbon-neutral means to generate hydrogen fuel from water, but the process typically requires expensive, rare metal catalysts. Here, the authors prepare hydrogen- and oxygen-evolving electrocatalysts from earth-abundant elements that outperform noble-metal counterparts.

## Introduction

The scalable storage of such abundant renewable energy sources as wind or solar energy is required to mitigate the aggravated global energy crisis while addressing the environmental issues^1^. Converting solar- or wind-derived electricity to hydrogen fuel via water electrolysis is an appealing means to accomplish this energy conversion and storage technology^2–6^. At present, there are mainly two commercialized water electrolysis including alkaline and proton exchange membrane (PEM) water electrolysis. PEM water electrolysis has high energy efficiency with high hydrogen production rate, but requires noble metal platinum (Pt) or iridium (Ir)-based catalysts^7,8^, making it unfavorable due to high cost and scarcity. The alternative, low-cost alkaline water electrolysis, is a mature technology for large-scale hydrogen production that is low-cost due to compatibility with non-noble catalysts, but it suffers from low production rates^9,10^. One of its grand challenges remains to the huge energy penalty caused by the uphill reaction kinetics of the catalysts that requires significantly high cell voltages (1.8–2.4 V, far larger than the thermodynamic value of 1.23 V) to catalyze the reaction with electrolysis currents of 200–400 mA cm^−2^, resulting in the production of less than 5% hydrogen by means of water electrolysis in the worldwide industry^10–12^. Therefore, it is urgent to rationally develop exceptionally efficient non-noble catalysts for expediting overall water splitting toward large-scale commercialization at high current densities with low cell voltages.

Currently, there exist some intriguing bifunctional catalysts to negotiate the overall water splitting efficiently in alkaline electrolytes, including transition-metal oxides (e.g., MoO_2_, NiCoO_4_)^13–15^, layered double hydroxides (LDH) (e.g., NiFe LDHs)^2,16^, sulfides (e.g., NiCo_2_S_4_, MoS_2_/Ni_3_S_2_)^17,18^, selenides (e.g., NiSe)^19^, and phosphides (e.g., CoP_2_/reduced graphene oxide, Ni_5_P_4_)^20,21^. Unfortunately, most of them can operate only steadily at low current density (<20 mA cm^−2^), not to mention their low energy conversion efficiency at above 200 mA cm^−2^ required for commercial applications. These catalysts are far from being optimized for industrial scales^10,22^ probably arisen from the difficulty in integrating both the merits of hydrogen evolution reaction (HER) and oxygen evolution reaction (OER) electrocatalysts in a single bifunctional catalyst in the same electrolyte (either alkaline or acid). In this regard, constructing a single bifunctional catalyst with outstanding HER and OER activities simultaneously in the same electrolyte is urgently needed.

We report here, just such a cost-effective catalyst that we discover using the straightforward strategy of hybridizing two metallic iron and dinickel phosphides (FeP/Ni_2_P) on commercial nickel (Ni) foams. This produces an extremely active bifunctional electrocatalyst for both OER and HER outperforming most of the catalysts with similar function, and also exceptional overall water splitting surpassing commercial alkaline electrolyzers in 1 M KOH. Specifically, we corroborate that our FeP/Ni_2_P hybrid performs well for HER with catalytic performance (−14 mV to achieve −10 mA cm^−2^) as good as that of the state-of-the-art noble Pt catalyst (−57 mV), and also for OER with the lowest overpotential (154 mV to afford 10 mA cm^−2^) reported thus far, substantially outperforming the benchmark IrO_2_ (281 mV) and other reported robust OER catalysts. Furthermore, inspired by the excellent HER and OER activity, we integrated this bifunctional catalyst directly as both the anode and cathode electrodes in an alkaline electrolyzer, and demonstrate that a cell voltage of only 1.42 V can deliver 10 mA cm^−2^, and a cell voltage of 1.72 V is required to deliver 500 mA cm^−2^ with 40 h durability, far surpassing the performance of current industrial catalysts, which require 2.40 V for 400 mA cm^−2^.

## Results

### Electrocatalyst preparation and characterization

**Fig. 1:**
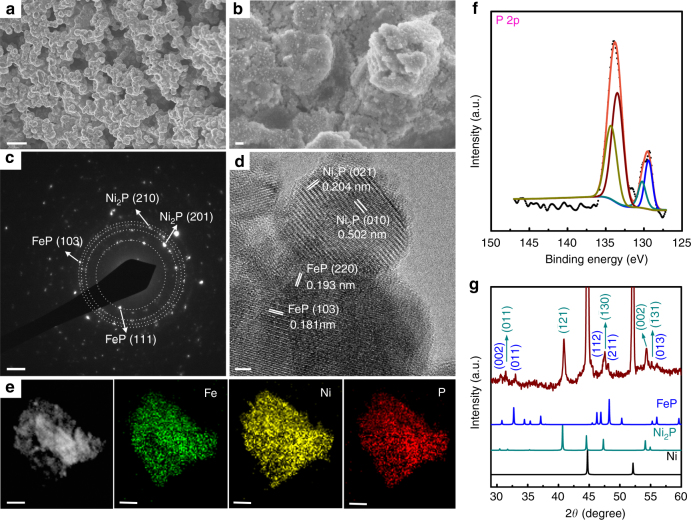
Synthesis and microscopic characterization of as-prepared FeP/Ni_2_P hybrid. **a** Low-magnification SEM images of FeP/Ni_2_P nanoparticles supported on Ni foam. Scale bar, 5 μm. **b** High-magnification SEM images of FeP/Ni_2_P nanoparticles supported on Ni foam. Scale bar, 200 nm. **c** The SAED pattern taken from the FeP/Ni_2_P catalysts. Scale bar, 2 1/nm. **d** A typical HRTEM image taken from the FeP/Ni_2_P catalysts. Scale bar, 2 nm. **e** The TEM image and corresponding EDX elemental mapping. Scale bar, 100 nm. **f** XPS analysis. **g** A typical XRD pattern of the samples (we did not show the full intensity of the peaks from Ni so that the peaks from the catalysts can be better viewed)

Our Fe–Ni–P hybrid architecture was prepared directly on commercial Ni foams by a simple thermal treatment process. Typical scanning electron microscopy (SEM) images show that the as-prepared samples are free-standing with abundant mesopores and/or nanopores at the surface (Fig. 1a and b), indicating efficacious achievement of large surface areas for facile exchange of proton or oxygen-containing intermediates^5,23^. In particular, numerous nanocrystals are distributed uniformly at the surface, forming plentiful surface active sites in this hybrid catalyst. The selected area electron diffraction pattern (Fig. 1c), combined with high-resolution transmission electron microscopy (TEM) images (Fig. 1d, Supplementary Fig. 1), further reveal the nanoscale features of the FeP and Ni_2_P particles with diameters of 5–30 nm. The interplanar spacings of these nanoparticles are resolved by TEM to be around 0.204 and 0.502 nm corresponding to the (021) and (010) planes of Ni_2_P crystals, and 0.181 and 0.193 nm corresponding to the (103) and (220) planes of FeP crystals. To determine the distribution of Ni, Fe, and P elements in the as-prepared samples, elemental mapping was carried out using TEM, confirming the homogenous distribution of Ni, Fe, and P elements in the FeP/Ni_2_P nanoparticles (Fig. 1e). The energy dispersive X-ray (EDX) spectrum (Supplementary Fig. 2) shows that the Ni, Fe, and P elements are present with an atomic ratio close to 2:1:2, consistent with the high-resolution TEM observations.

The chemical composition and oxidation states of the catalysts were further unveiled by X-ray photoelectron spectroscopy (XPS) and X-ray diffraction (XRD). The P 2p core level spectrum can be fit with two doublets (Fig. 1f), with one located at the binding energies of 129.3 and 130.1 eV attributing to phosphorus anions of metal phosphides, and the other at 133.5 and 134.3 eV indicative of phosphate-like P arisen from possible surface oxidation, as has been observed previously^24–26^. The XPS spectrum of Fe 2p^3/2^ core level (Supplementary Fig. 3a) can be deconvoluted into three main peaks with binding energies of 707.0, 709.9, and 711.9 eV assigned to FeP, Fe-based oxide, and phosphate, respectively, caused by possible superficial oxidation when exposing FeP samples to air^26–28^, while another peak located at 714.3 eV is arisen from the relevant satellite peak. This peak deconvolution is also applied to the Ni 2p^3/2^ core level spectrum (Supplementary Fig. 3b), where three binding energies located at 853.6, 856.4, and 861.0 eV are ascribed to Ni_2_P, Ni-PO_*x*_, and the corresponding satellite peak, respectively. These information means that both the FeP and Ni_2_P contribute to the overall signals with the binding energy at 129.3 eV of P 2p^3/2^, even though it is difficult to distinguish the binding energy difference between these two compounds. According to the survey spectrum (Supplementary Fig. 4) and distribution quantification (Supplementary Table 1), it is estimated that the percentage of surface oxidized species in FeP nanoparticles is close to 74.8%, and the percentage in Ni_2_P is around 13.5%, which indicates that the original Fe–Ni–P samples are heavily oxidized at the surface. A typical XRD pattern (Fig. 1g) reveals the main indexes from the as-prepared FeP/Ni_2_P hybrid and Ni foam support. The two strongest peaks at 45° and 52° are mainly originated from the Ni foam support (ICSD-53809). All the other peaks are the characteristic ones of FeP (ICSD-633046) and Ni_2_P (ICSD-646102), consistent with our TEM analysis.

### Oxygen evolution catalysis

**Fig. 2:**
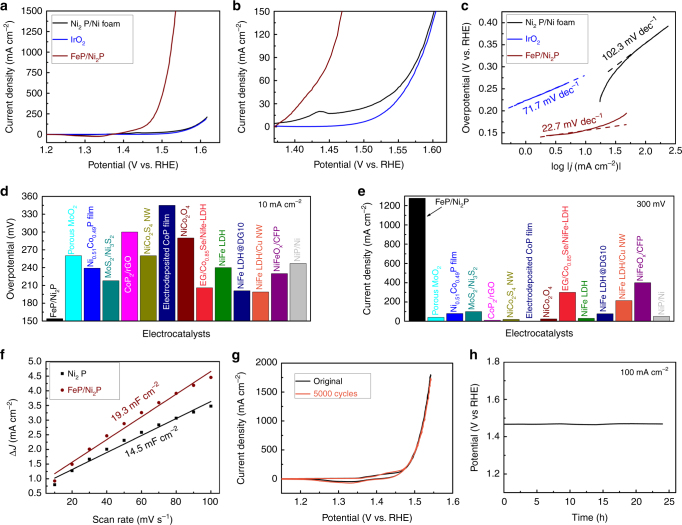
Electrocatalytic oxygen evolution reaction. **a** The polarization curves recorded on different catalysts. **b** The enlarged region of the curves in **a**. **c** The corresponding Tafel plots. **d** Comparison of the overpotentials required at 10 mA cm^−2^ among our catalyst and available reported OER catalysts. **e** Comparison of the current densities delivered at 300 mV among our catalyst and available reported OER catalysts. **f** Double-layer capacitance (*C*_dl_) measurements of Ni_2_P and FeP/Ni_2_P catalysts. **g** Cyclic voltammetry (CV) curves of FeP/Ni_2_P before and after the acceleration durability test for 5000 cycles. **h** Time-dependent potential curve for FeP/Ni_2_P at 100 mA cm^-2^. Electrolyte: 1 M KOH

We first evaluated the catalytic OER activity of this Fe–Ni–P hybrid catalyst in 1.0 M KOH electrolyte^6,29^. Representative polarization curves in Fig. 2a and b show the geometric current density plotted against applied potential vs reversible hydrogen electrode (RHE) of this Fe–Ni–P hybrid electrode relative to Ni_2_P and benchmark IrO_2_ catalysts. The effect of capacitive current on the catalytic activity, originating from the Ni ions oxidation, is minimized by calculating the average activity from the forward and backward sweeps of a cyclic voltammetry (CV) curve (Supplementary Fig. 5)^30,31^. Strikingly, the Fe–Ni–P hybrid requires an overpotential of only 154 mV to deliver 10 mA cm^−2^, which is 127 mV less than the state-of-the-art IrO_2_ catalyst (281 mV). At 281 mV, our FeP/Ni_2_P catalyst achieves a current density up to 690 mA cm^−2^, which is 69-fold higher than the benchmark IrO_2_, demonstrating a huge improvement of the OER activity. Indeed this overpotential of 154 mV in alkaline conditions is among the lowest for catalyzing OER thus far (Supplementary Table 2), even surpassing the presently most active NiFe LDH (double layered hydroxide) catalyst (~200 mV)^16,32^. We measured a very small Tafel slope of 22.7 mV dec^−1^ in the low overpotential ranges^33^ (Fig. 2c, Supplementary Fig. 6), which is much smaller than those of the reference materials Ni_2_P (102.3 mV dec^−1^) and IrO_2_ (71.7 mV dec^−1^), and also smaller than most of the OER catalysts reported (Supplementary Table 2). Specifically, we further compared the OER activity with other available bifunctional catalysts as shown in Fig. 2d and e. It is evident that our catalyst requires the lowest overpotential 154 mV to achieve 10 mA cm^−2^, and very large current density (1277 mA cm^−2^) at 300 mV overpotential, indicating the potential to be used for overall water splitting with large current densities at small cell voltage.

To elucidate the origins of this remarkably high OER catalytic activity, we performed electrochemical impedance spectroscopy (EIS) and double-layer capacitance (*C*_dl_) investigations on this FeP/Ni_2_P electrode. This capacitance *C*_dl_ determined by a simple CV method^5,29,34–36^ (Supplementary Fig. 7) is calculated to be 19.3 mF cm^−2^ for the Fe–Ni–P hybrid electrode (Fig. 2f), very close to that of the Ni_2_P catalyst (14.5 mF cm^−2^). This manifests that depositing FeP on the Ni_2_P surface does not result in huge changes in the active surface area, while the electrochemical OER performance of FeP/Ni_2_P is much better than Ni_2_P. For instance, our FeP/Ni_2_P hybrid achieves 1000 mA cm^−2^ at 293 mV, while Ni_2_P can deliver only 32 mA cm^−2^ at this overpotential, making our FeP/Ni_2_P catalyst ~30-fold better than the Ni_2_P catalyst, heralding that synergistic effects between FeP and Ni_2_P in the hybrid is the main contributor to our superior catalytic performance, not just the high active surface area. Meanwhile, the EIS spectra show that this FeP/Ni_2_P hybrid has a lower charge-transfer resistance at the interface of the catalysts with Ni foam, leading to faster OER kinetics compared to the Ni_2_P catalyst (Supplementary Fig. 8). Additionally, according to our simulations, both the FeP(001)/Ni_2_P and FeP(010)/Ni_2_P have electrons transferred from Ni_2_P to FeP at neutral with 0.15 C m^−2^ and 0.097 C m^−2^, or 0.075e^−^and 0.051e^−^ per surface Ni, respectively. Since electrons are depleted on Ni_2_P, holes are created and the Fermi level on Ni_2_P is shifted downwards, which facilitates the OER process. Especially, the charge transfers in the presence of 2, 3, and 4 layers of FeP on Ni_2_P were further calculated to understand the effect of different loadings of FeP on OER activities. The corresponding values are 0.009e^−^, 0.075e^−^, and 0.078e^−^ per surface Ni, respectively. The result of converging charge transfers in the presence of more FeP implies that there is no more advantage in charge transfer beyond a certain amount of FeP is used, which leads to similar activities. Furthermore, the catalytic OER performances were compared by growing additional Ni_2_P particles, FeP particles with different catalyst loadings on top of Ni foam (Supplementary Figs. 9–11). It is obvious that increasing the loading of Ni_2_P particles has very few effects on improving the OER performances, and an oxidation peak regarding the Ni^II^ to Ni^III^ becomes stronger with the increase of Ni_2_P loadings (Supplementary Fig. 9). Once FeP particles were grown atop the Ni_2_P/Ni foam, the performance can be greatly enhanced due to the following reasons: the Ni_2_P/Ni support has good conductivity and high surface area for growing uniform FeP particles in small size with enhanced OER activity, the oxidation peak of Ni^II^ to Ni^III^ can be weakened by FeP modification (Supplementary Fig. 9–11), and many Fe impurities are possibly provided to improve the catalytic activity of underlying Ni_2_P catalyst by Fe incorporation^37^. All these information confirms strong synergistic effects between FeP and Ni_2_P particles. Finally, it is noted that there are a large amount of oxidized species at the surface of original FeP/Ni_2_P (Supplementary Fig. 3). These surface oxidized species may play a positive role in the OER activity according to recent studies^38–40^. It is possible that they act as a labile ligand to vary the coordination or chelating modes during the redox switching process, and also facilitates the 4e multiproton-coupled electron transfers step in the OER process. The formation of metal phosphide–metal oxide interface may also be helpful to the efficient carrier transportation from the phosphide core to the oxidized species^40^. Therefore, we attributed the excellent OER activity of our FeP/Ni_2_P hybrid catalyst to the presence of surface oxidized species, fast electron transport, and synergistic effects between FeP and Ni_2_P.

Electrochemical durability is another key index to evaluate the performance of electrocatalysts. Obviously, after 5000 cycling test, the CV curve of this FeP/Ni_2_P hybrid is nearly identical to the original one, suggesting its excellent durability during cycling scans (Fig. 2g). We also probed the long-term electrochemical stability of the catalyst tested at 100 mA cm^−2^, finding that the real-time potential remains nearly constant during a 24 h continuous operation (Fig. 2h). These results establish the strong durability of FeP/Ni_2_P catalyst for OER in alkaline electrolyte. Further insights into the chemical compositions for post-OER samples by XPS (Supplementary Fig. 12) and XRD (Supplementary Fig. 13) confirm that a mixture of nickel and iron oxides/oxyhydroxides evolves at the surface of the FeP/Ni_2_P hybrid, possibly acting as the real active sites for OER^6^. This behavior is also observed for many metal sulfides^41^, phosphides^42^, or selenides^19^, which are easily oxidized to oxides or hydroxides under the OER process at high anodic potentials.

### Hydrogen evolution catalysis

**Fig. 3:**
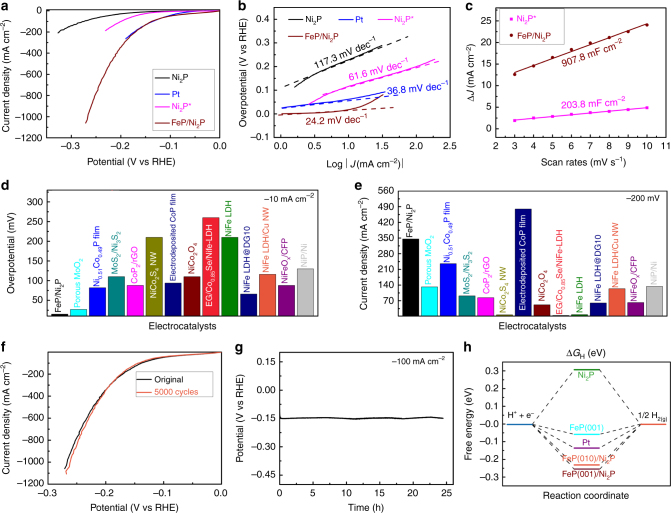
Electrocatalytic hydrogen evolution reaction. **a** The HER polarization curves of different catalysts. **b** The relevant Tafel plots. **c** Double-layer capacitance measurements for determining electrochemically active surface areas of Ni_2_P and FeP/Ni_2_P electrodes. **d** Comparison of the overpotentials required at 10 mA cm^−2^ among our catalyst and available reported HER catalysts. **e** Comparison of the current densities delivered at −200 mV among our catalyst and available reported HER catalysts. **f** Polarization curves before and after 5000 cycling test. **g** The chronopotentiometric curve of the FeP/Ni_2_P electrode tested at a constant current density of −100 mA cm^−2^ for 24 h. **h** Free energy diagram for ∆*G*_H_, the hydrogen adsorption free energy at pH = 14 on FeP/Ni_2_P catalyst in comparison with Ni_2_P and benchmark Pt catalysts. Electrolyte: 1 M KOH

In addition to the excellent OER performance, we found that this FeP/Ni_2_P hybrid is highly active towards HER in the same electrolyte. It is evident that the bare Ni_2_P is not a good HER catalyst requiring a large overpotential of 150 mV to deliver a current density of −10 mA cm^−2^ (Fig. 3a, Supplementary Fig. 14). Distinctly, our FeP/Ni_2_P hybrid obtains −10 mA cm^−2^ at an extremely low overpotential of 14 mV, which is the lowest value among non-noble metal-based HER catalysts (Supplementary Table 3), and indeed is comparable to that of Pt (59 mV) in alkaline electrolyte. Meanwhile, the Tafel slope of this FeP/Ni_2_P catalyst is only 24.2 mV dec^−1^ in the low overpotential ranges (Fig. 3b, Supplementary Fig. 15), which is lower than that of Ni_2_P (117.3 mV dec^−1^) and Pt (36.8 mV dec^−1^). To gain further insight into the outstanding HER activity, the *C*_dl_ values (Fig. 3c, Supplementary Fig. 16) were utilized to compare the active surface area, confirming that both high active surface area (907.8 mF cm^−2^) and small charge-transfer resistance (Supplementary Fig. 17) contribute greatly to the outstanding HER catalytic activity of this FeP/Ni_2_P hybrid^5,23^. It is noted that the capacitance is different when the same FeP/Ni_2_P catalyst was used for HER and OER, which is possibly due to the different origins of active sites for the OER and HER. In particular, we prepared pure Ni_2_P^*^ catalyst on Ni foam with similar mass loading in the same growth conditions as FeP/Ni_2_P. In this case, we found that Ni_2_P^*^ catalyst still shows catalytic activity inferior to the FeP/Ni_2_P hybrid, and has a smaller *C*_dl_ value (Fig. 3c). After normalizing the polarization curves by the active surface area or *C*_dl_ difference, the FeP/Ni_2_P hybrid still exhibits better catalytic HER activity than pure Ni_2_P^*^ catalyst (Supplementary Fig. 18), meaning that FeP/Ni_2_P has better intrinsic activity than pure Ni_2_P^*^ catalyst. Then the intrinsic catalytic activity was assessed by the turnover frequency (TOF) for each active site quantified by an electrochemical method^43^ (Supplementary Note 1, Supplementary Fig. 19). From this method, the number of active catalytic sites for the FeP/Ni_2_P hybrid is around 2.5 times that of the Ni_2_P^*^ catalyst, and accordingly the TOF of the FeP/Ni_2_P hybrid is calculated to be 0.163 s^−1^ at 100 mV overpotential, which is much higher than that of pure Ni_2_P^*^ catalyst (0.006 s^−1^) at the same overpotential, suggesting that the addition of FeP particles on Ni_2_P surface contributes more to the improvement of the HER activities of this FeP/Ni_2_P hybrid. This is further supported by measuring the catalytic activities of additional Ni_2_P or FeP particles with different loadings grown on Ni_2_P/Ni support (Supplementary Fig. 9–11). More interestingly, this FeP/Ni_2_P hybrid still shows outstanding HER activity compared to other available bifunctional catalysts (Fig. 3d and e). To evaluate its stability during electrochemical HER, a long-term cycling test (Fig. 3f) and continuous operation for 24 h of hydrogen release at −100 mA cm^−2^ (Fig. 3g) were performed in 1 M KOH, demonstrating its good stability.

In order to figure out the factors contributing to the superior HER activity, we performed density functional theory (DFT) calculations on this catalyst. According to Fig. 1d, (021) and (010) lattice planes are observed on Ni_2_P nanoparticles. Since (021) and (010) of Ni_2_P have a simple common perpendicular direction (100), we chose this plane to model Ni_2_P (Supplementary Fig. 20). On the other hand, the two directions, (220) and (103) on FeP, do not share a common simple perpendicular direction, hence we chose two different directions, (001) and (010), to model FeP. Since the overall system involved two materials, the interactions between FeP and Ni_2_P were modeled by placing FeP on top of Ni_2_P, which is reasonable since an Ni foam was used as the material on which Ni_2_P and FeP were grown. The corresponding lattice distances were chosen to minimize the percent changes in both Ni_2_P and FeP. The hydrogen adsorption energy, ∆*G*_H_, was calculated in the same way as in our previous study^5^ and is shown in Supplementary Table 4. As shown in Fig. 3h, Supplementary Fig. 20 and Supplementary Table 4, pure Ni_2_P (001) leads to a relatively strong exothermic ∆*G*_H_ (0.306 eV), indicating that it is not the most active center for the hydrogen evolution electrocatalysis, which we confirmed experimentally (Fig. 3a, b, Supplementary Fig. 18). However, this hydrogen adsorption energy │∆*G*_H_│ is reduced significantly to 0.255 and 0.230 eV for a very thin FeP (100) or FeP (010) crystal (~3 layers), respectively, hybridized atop with Ni_2_P. It is worth pointing out that we confine our calculation a thin layer of FeP crystal, ignoring the particulate size (5–30 nm), so we hypothesized that the as-synthesized FeP nanoparticles along with Ni_2_P preferentially expose the most active facets as those of bulk FeP (001) crystal, which results in further reduction of │∆*G*_H_│ to only 0.06 eV, contributing to the high activity not seen in typical FeP crystals. This conclusion is also supported by the above experiments regarding the TOF calculation. Thus, both the experiment and theory support that this hybrid catalyst is an efficient HER electrocatalyst.

### Overall water splitting

**Fig. 4:**
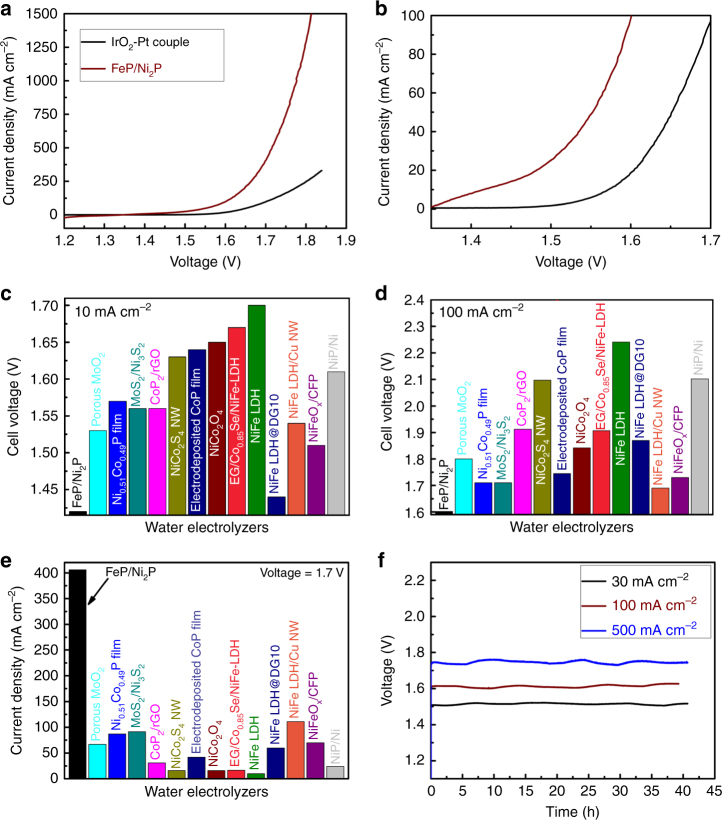
Overall-water-splitting activity of the FeP/Ni_2_P catalyst. **a** The polarization curve of FeP/Ni_2_P and IrO_2_-Pt coupled catalysts in a two-electrode configuration. **b** Enlarged version at low current density region of **a**. **c** Comparison of the cell voltages to achieve 10 mA cm^−2^ among different water alkaline electrolyzers. **d** Comparison of the cell voltages to achieve 100 mA cm^−2^ among different water alkaline electrolyzers. **e** Comparison of the current densities at 1.7 V for this FeP/Ni_2_P catalyst with available non-noble bifunctional catalysts. **f** Catalytic stability of the FeP/Ni_2_P catalysts at 30, 100, and 500 mA cm^−2^ for around 40 h. Electrolyte: 1 M KOH

Given the outstanding OER and HER activities in 1 M KOH electrolyte, we further utilized this FeP/Ni_2_P hybrid as both anode and cathode in a two-electrode configuration for overall water splitting in the same electrolyte. Remarkably, the cell voltage to afford a current density of 10 mA cm^−2^ is as low as 1.42 V with a relatively low Tafel slope of 69.5 mV dec^−1^ (Fig. 4a, b, Supplementary Figs. 21 and 22), substantially lower than that of the coupled benchmark IrO_2_-Pt catalysts (1.57 V), and superior to most previously reported bifunctional electrocatalysts, which generally need cell voltages higher than 1.50 V to deliver the same current density (Fig. 4c, Supplementary Table 5). This cell voltage also manifests that the electrical-to-fuel efficiency of water-splitting electrolyzers at 10 mA cm^−2^ is dramatically elevated to 86.6% using only this material, making it of great potential for scale-up water electrolysis with high efficiency and low cost. Even though the best bifunctional NiFe LDH catalyst reported recently can deliver 20 mA cm^−2^ at a cell voltage of 1.50 V, which is close to our FeP/Ni_2_P catalyst (1.48 V), but a much larger cell voltage of 1.70 V is needed to achieve only 60 mA cm^−2^, meaning low energy conversion efficiency at high current density^16^. Nearly all the bifunctional electrocatalysts require larger than 1.69 V to reach 100 mA cm^−2^ for the overall water splitting (Fig. 4d). Even at 1.7 V cell voltage, most of the electrolyzers can only deliver current densities below 110 mA cm^−2^ (Fig. 4e). In contrast, our FeP/Ni_2_P catalyst can readily drive water electrolysis at high current densities of 100, 500, and 1000 mA cm^−2^ at very low cell voltages of 1.60, 1.72, and 1.78 V, respectively, showing that our catalyst performs outstandingly over the full range of current density. We further tested the long-term stability of our FeP/Ni_2_P electrode at 30 and 100 mA cm^−2^ for 36 h, showing no detectable voltage decay (Fig. 4f). Moreover, we further examined extremely high-current operation of the electrolyzer at 1.72 V for overall water splitting at 500 mA cm^−2^, which is a big step toward real industrial applications^6,22^. In comparison, commercial alkaline water electrolysis^10,15^ requires 1.8–2.4 V to generate 200–400 mA cm^−2^, while no previous bifunctional catalysts show catalytic activities superior to the commercial ones with good durability at high current density above 200 mA cm^−2^. In contrast, our alkaline electrolyzer only requires 1.72 V to afford 500 mA cm^−2^ while also exhibiting excellent stability for more than 40 h confirmed by steady chronopotentiometric testing (Fig. 4f). Especially, using the gas chromatography-based technique^5^, we found that H_2_ and O_2_ are the only gas products during water electrolysis, and the molar ratio between H_2_ and O_2_ is close to 2:1 (Supplementary Fig. 23), suggesting that nearly all the electrons are actively involved in the catalytic reaction. This demonstrates outstanding overall-water-splitting activity of our hybrid catalyst, making it an excellent condition for industrial use.

## Discussion

In summary, we developed an FeP/Ni_2_P hybrid catalyst supported on 3D Ni foam that proves to be an outstanding bifunctional catalyst for overall water splitting, exhibiting both extremely high OER and HER activities in the same alkaline electrolyte. Indeed, it requires a very low cell voltage of 1.42 V to afford 10 mA cm^−2^ in alkaline water electrolyzers, while at the commercially practical current density of 500 mA cm^−2^, it demands only a voltage of 1.72 V lower than those for any reported bifunctional catalysts, and maintains its excellent catalytic activity for more than 40 h at a current density of 500 mA cm^−2^, paving the way for promising large-scale hydrogen generation.

## Methods

### Chemicals

Red phosphorous powder (Sigma-Aldrich, ≥97%, CAS No. 7723-14-0), Iron(III) nitrate nonahydrate [Sigma-Aldrich, Fe(NO_3_)_3_·9H_2_O, ≥99.95%, CAS No. 7782-61-8], Pt wire (CH Instruments, Inc.), Nafion 117 solution (5%; Sigma-Aldrich), iridium oxide powder (Alfa Aesar, IrO_2_, 99%), potassium hydroxide (Alfa Aesar, KOH, 50% wt/vol), and Ni foam (areal density 320 g cm^−2^) (ref. ^2^) were used without further purification.

### Material synthesis

These metal phosphides (Ni_2_P and FeP) were grown by chemical vapor deposition in a tube furnace, in which Ni foam, Fe(NO_3_)_3_, and phosphorus were utilized as the Ni, Fe, and P sources, respectively. Namely, we first immersed a commercially hydrophobic Ni foam into an aqueous Fe(NO_3_)_3_ solution (0.37 M), which was then converted to mainly Ni_2_P and a very small fraction of FeP at 450 °C in Ar atmosphere, with phosphorus powder supplied upstream. After that, the samples were naturally cooled down under Ar gas protection, which became hydrophilic after first phosphidation. In the following, a second-time phosphidation was performed after the samples were immersed into the Fe(NO_3_)_3_ solution again. The catalyst loading is around 8 mg cm^−2^ for the FeP/Ni_2_P catalyst. For comparison, the as-prepared Ni_2_P samples were obtained in the same growth conditions without the addition of Fe(NO_3_)_3_, and the Ni_2_P^*^ samples were grown in the same experimental conditions by using Ni(NO_3_)_2_ (0.37 M) instead of Fe(NO_3_)_3_. Three different concentrations of Fe(NO_3_)_3_ (0.25, 0.37, and 0.50 M) and Ni(NO_3_)_2_ (0.17, 0.37, and 0.50 M) precursors were prepared to grow different catalyst loadings of FeP (6.0, 8.0, and 12.5 mg cm^−2^) or Ni_2_P particles (3.0, 7.5, and 10.5 mg cm^−2^) on the surface, respectively, so as to optimize the experimental conditions and relevant catalytic activities for the HER and OER. The optimal loading of FeP/Ni_2_P hybrid catalysts was found to be around 8 mg cm^−2^ on Ni foam.

### Electrochemical characterization

The electrochemical tests were performed via a typical three-electrode configuration in 100 ml 1 M KOH electrolyte^6,29^. The polarization curves for the HER were recorded by linear sweep voltammetry with a scan rate of 1.0 mV s^−1^. For the OER and overall water splitting, in order to minimize the effect of capacitive current originating from the Ni ions oxidation on the catalytic performance, CV curves with the forward and backward sweeps with a very small scan rate of 1 mV s^−1^ were utilized to calculate the average activity. A carbon paper was used as the counter electrode for both the HER and OER tests. The scan rate for the cycling tests was set to 50 mV s^−1^. All the potentials shown here were converted to RHE.

### Computational methods

GGA level of DFT was employed to calculate the relative energies of relevant structures in this study. More specifically, PBE^44,45^ functional with the D3 ^46^ correction was used for both geometry optimizations and the single point free energies. Geometry optimizations were performed in VASP^47,48^ with projected augmented wave (PAW)^49,50^ and VASPsol^51^ solvation. The kinetic energy cutoff was 300 eV for geometry optimization, and 13 Hartree (354 eV) for single point energy. The single point free energies were calculated in jDFTx^52–56^ with CANDLE^57^ implicit solvation and GBRV uspp pseudopotentials. The final free energy *G* was calculated as *G* = *F* − *n*_e_*U* + ZPE + *H*_vib_ − *TS*_vib_, where *F* is the energy of the solvated Kohn–Sham DFT electronic system, *n*_e_ is the number of electrons, and *U* is the chemical potential for the electrons. Also, in order to understand the charge transfer process between Ni_2_P and FeP, we calculated the Mulliken charges of the hybrid FeP/Ni_2_P structure and summed up the charges for each compound. Since Mulliken charges can only be rigorously defined using localized basis functions, we performed this set of DFT calculations on the same structures used in the HER part of the study using Gaussian basis functions in CRYSTAL14 with PBE and the same k-point grid.

### Data availability

The data that support the findings of this work are available from the corresponding author upon reasonable request.

## Electronic supplementary material

Supplementary Information

## References

[1] Zou XX, Zhang Y (2015). Noble metal-free hydrogen evolution catalysts for water splitting. Chem. Soc. Rev..

[2] Luo JS (2014). Water photolysis at 12.3% efficiency via perovskite photovoltaics and Earth-abundant catalysts. Science.

[3] Wang XG, Kolen’ko YV, Bao XQ, Kovnir K, Liu LF (2015). One-step synthesis of self-supported nickel phosphide nanosheet array cathodes for efficient electrocatalytic hydrogen generation. Angew. Chem. Int. Ed..

[4] Faber MS, Jin S (2014). Earth-abundant inorganic electrocatalysts and their nanostructures for energy conversion applications. Energy Environ. Sci..

[5] Zhou HQ (2016). Efficient hydrogen evolution by ternary molybdenum sulfoselenide particles on self-standing porous nickel diselenide foam. Nat. Commun..

[6] Zhou HQ (2017). Highly active catalyst derived from a 3D foam of Fe(PO_3_)_2_/Ni_2_P for extremely efficient water oxidation. Proc. Natl. Acad. Sci. USA.

[7] Ng JWD (2016). Gold-supported cerium-doped NiO_x_ catalysts for water oxidation. Nat. Energy.

[8] Cheng NC (2016). Platinum single-atom and cluster catalysis of the hydrogen evolution reaction. Nat. Commun..

[9] Leng Y (2012). Solid-state water electrolysis with an alkaline membrane. J. Am. Chem. Soc..

[10] Zeng K, Zhang DK (2010). Recent progress in alkaline water electrolysis for hydrogen production and applications. Prog. Energy. Combust. Sci..

[11] Carmo M, Fritz DL, Merge J, Stolten D (2013). A comprehensive review on PEM water electrolysis. Int. J. Hydrog. Energy.

[12] Sun Y, Delucchi M, Ogden J (2011). The impact of widespread deployment of fuel cell vehicles on platinum demand and price. Int. J. Hydrog. Energy.

[13] Jin YS (2016). Porous MoO_2_ nanosheets as non-noble bifunctional electrocatalysts for overall water splitting. Adv. Mater..

[14] Gao XH (2016). Hierarchical NiCo_2_O_4_ hollow microcuboids as bifunctional electrocatalysts for overall water splitting. Angew. Chem. Int. Ed..

[15] Wang HT (2015). Bifunctional non-noble metal oxide nanoparticle electrocatalysts through lithium-induced conversion for overall water splitting. Nat. Commun..

[16] Jia Y (2017). A heterostructure coupling of exfoliated Ni-Fe hydroxide nanosheet and defective graphene as a bifunctional electrocatalyst for overall water splitting. Adv. Mater..

[17] Zhang J (2016). Interface engineering of MoS_2_/Ni_3_S_2_ heterostructures for highly enhanced electrochemical overall-water-splitting activity. Angew. Chem. Int. Ed..

[18] Sivanantham A, Ganesan P, Shanmugam S (2016). Hierarchical NiCo_2_S_4_ nanowire arrays supported on Ni foam: an efficient and durable bifunctional electrocatalyst for oxygen and hydrogen evolution reactions. Adv. Funct. Mater..

[19] Tang C, Cheng NY, Pu ZH, Xing W, Sun XP (2015). NiSe nanowire film supported on nickel foam: an efficient and stable 3D bifunctional electrode for full water splitting. Angew. Chem. Int. Ed..

[20] Wang JM, Yang WR, Liu JQ (2016). CoP_2_ nanoparticles on reduced graphene oxide sheets as a super-efficient bifunctional electrocatalyst for full water splitting. J. Mater. Chem. A.

[21] Ledendecker M (2015). The synthesis of nanostructured Ni_5_P_4_ films and their use as a non-noble bifunctional electrocatalyst for full water splitting. Angew. Chem. Int. Ed..

[22] Smith RDL (2013). Photochemical route for accessing amorphous metal oxide materials for water oxidation catalysis. Science.

[23] Cabán-Acevedo M (2015). Efficient hydrogen evolution catalysis using ternary pyrite-type cobalt phosphosulphide. Nat. Mater..

[24] Yang Y, Fei HL, Ruan GD, Tour JM (2015). Porous cobalt-based thin film as a bifunctional catalyst for hydrogen generation and oxygen generation. Adv. Mater..

[25] Zhu YP, Liu YP, Ren TZ, Yuan ZY (2015). Self-supported cobalt phosphide mesoporous nanorod arrays: a flexible and bifunctional electrode for highly active electrocatalytic water reduction and oxidation. Adv. Funct. Mater..

[26] Xu JY, Xiong DH, Amorim I, Liu LF (2018). Template-free synthesis of hollow iron phosphide-phosphate composite nanotubes for use as active and stable oxygen evolution electrocatalysts. ACS Appl. Nano Mater..

[27] Tian JQ (2014). FeP nanoparticles film grown on carbon cloth: an ultrahighly active 3D hydrogen evolution cathode in both acidic and neutral solutions. ACS Appl. Mater. Interfaces.

[28] Yan Y (2015). A flexible electrode based on iron phosphide nanotubes for overall water splitting. Chem. Eur. J..

[29] Yu F (2017). Three-dimensional nanoporous ion nitride film as an efficient electrocatalyst for water oxidation. ACS Catal..

[30] Stevens MB (2017). Measurement techniques for the study of thin film heterogeneous water oxidation electrocatalysts. Chem. Mater..

[31] Liang HW (2015). Molecular metal-N_x_ centres in porous carbon for electrocatalytic hydrogen evolution. Nat. Commun..

[32] Li ZH (2015). Fast electrosynthesis of Fe-containing layered double hydroxide arrays toward highly efficient electrocatalytic oxidation reactions. Chem. Sci..

[33] Shinagawa T, Garcia-Esparza AlT, Takanabe K (2015). Insight on Tafel slopes from a microkinetic analysis of aqueous electrocatalysis for energy conversion. Sci. Rep..

[34] Zhou HQ (2016). Highly efficient hydrogen evolution from edge-oriented WS_2(1-x)_Se_2x_ particles on three-dimensional porous NiSe_2_ foam. Nano Lett..

[35] Kong DS, Wang HT, Lu ZY, Cui Y (2014). CoSe_2_ nanoparticles grown on carbon fiber paper: an efficient and stable electrocatalyst for hydrogen evolution reaction. J. Am. Chem. Soc..

[36] Mishra IK (2018). Highly efficient hydrogen evolution by self-standing nickel phosphide-based hybrid nanosheet arrays electrocatalyst. Mater. Today Phys..

[37] Trotochaud L, Young SL, Ranney JK, Boettcher SW (2014). Nickel-iron oxyhydroxide oxygen-evolution electrocatalysts: the role of intentional and incidental iron incorporation. J. Am. Chem. Soc..

[38] Ahn HS, Tilley TD (2013). Electrocatalytic water oxidation at neutral pH by a nanostructured Co(PO_3_)_2_ anode. Adv. Funct. Mater..

[39] Chang JF (2015). Surface oxidized cobalt-phosphide nanorods as an advanced oxygen evolution catalyst in alkaline solution. ACS Catal..

[40] Anirban D, Pradhan N (2017). Developments of metal phosphides as efficient OER precatalysts. J. Phys. Chem. Lett..

[41] You B, Sun YJ (2016). Hierarchically porous nickel sulfide multifunctional superstructures. Adv. Energy Mater..

[42] Stern LA, Feng LG, Song F, Hu XL (2015). Ni_2_P as a Janus catalyst for water splitting: the oxygen evolution activity of Ni_2_P nanoparticles. Energy Environ. Sci..

[43] Merki D, Fierro S, Vrubel H, Hu XL (2011). Amorphous molybdenum sulfide films as catalysts for electrochemical hydrogen production in water. Chem. Sci..

[44] Perdew JP, Burke K, Ernzerhof M (1996). Generalized gradient approximation made simple. Phys. Rev. Lett..

[45] Perdew JP, Burke K, Ernzerhof M (1997). K+ emission in symmetric heavy ion reactions at subthreshold energies. Phys. Rev. Lett..

[46] Grimme S, Antony J, Ehrlich S, Krieg S (2010). A consistent and accurate ab initio parametrization of density functional dispersion correction (DFT-D) for the 94 elements H-Pu. J. Chem. Phys..

[47] Kresse G, Hafner J (1993). Ab initio molecular dynamics for liquid metals. Phys. Rev. B Condens. Matter Mater. Phys..

[48] Kresse G, Furthmuller J (1996). Efficient iterative schemes for ab initio total-energy calculations using a plane-wave basis set. Phys. Rev. B Condens. Matter Matter. Phys..

[49] Blochl PE (1994). Projector augmented-wave method. Phys. Rev. B Condens. Matter Mater. Phys..

[50] Kresse G, Joubert D (1999). From ultrasoft pseudopotentials to the projector augmented-wave method. Phys. Rev. B Condens. Matter Mater. Phys..

[51] Mathew K, Sundararaman R, Letchworth-Weaver K, Arias TA, Hennig RG (2014). Implicit solvation model for density-functional study of nanocrystal surfaces and reaction pathways. J. Chem. Phys..

[52] Ismail-Beigi S, Arias TA (2000). New algebraic formulation of density functional calculation. Comput. Phys. Commun..

[53] Rozzi CA, Varsano D, Marini A, Gross EKU, Rubio A (2006). Exact Coulomb cutoff technique for supercell calculations. Phys. Rev. B.

[54] Petrosyan SA, Rigos AA, Arias TA (2005). Joint density-functional theory: ab initio study of Cr_2_O_3_ surface chemistry in solution. J. Phys. Chem. B.

[55] Arias TA, Payne MC, Joannopoulos JD (1992). Ab initio molecular dynamics: analytically continued energy functionals and insights into iterative solutions. Phys. Rev. Lett..

[56] Freysoldt C, Boeck S, Neugebauer J (2009). Direct minimization technique for metals in density functional theory. Phys. Rev. B.

[57] Sundararaman R, Goddard WA (2015). The charge-asymmetric nonlocally determined local-electric (CANDLE) solvation model. J. Chem. Phys..

