# Supplementary material for: High-performance bifunctional porous non-noble metal phosphide catalyst for overall water splitting
Source: Nat Commun. 2018 Jun 29;9:2551. doi: 10.1038/s41467-018-04746-z (PMC6026163; doi:10.1038/s41467-018-04746-z)
Supplement: Supplementary file 1 — Supplementary Information [file 41467_2018_4746_MOESM1_ESM.pdf]

Supplementary Information for

High-performance bifunctional porous non-noble metal phosphide catalyst for overall water splitting

Yu *et al*.

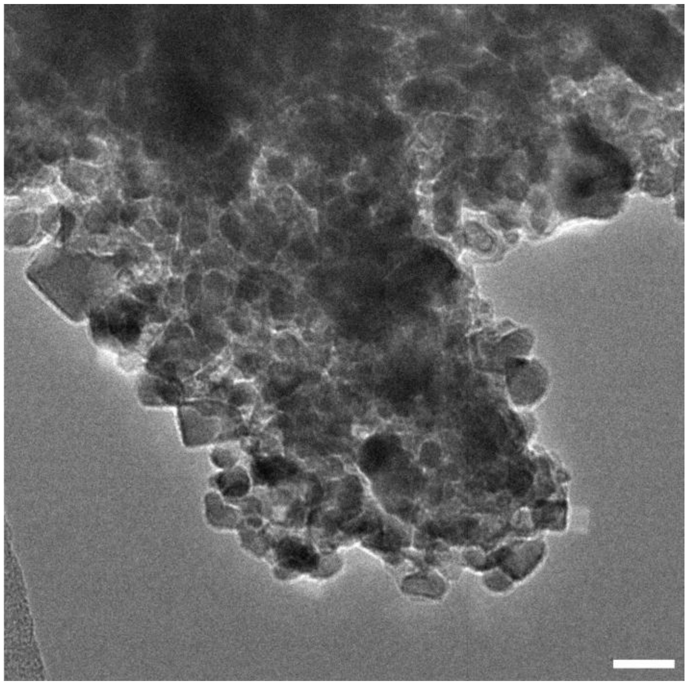

Supplementary Figure 1. A typical TEM image of as-prepared FeP/Ni_2P nanoparticles. Scale bar: 25 nm.

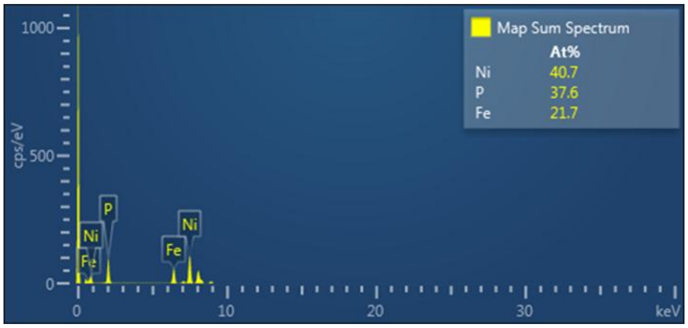

Supplementary Figure 2. Energy dispersive X-ray (EDX) spectrum of FeP/Ni_2P nanoparticles.

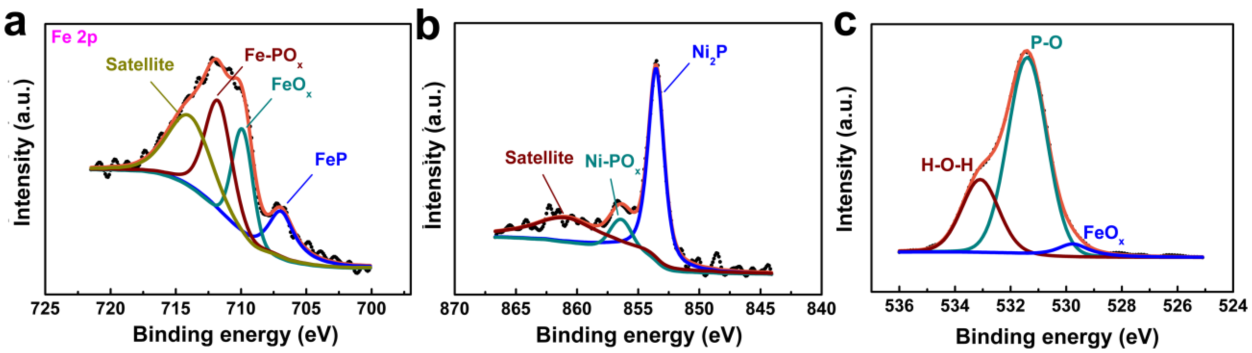

Supplementary Figure 3. XPS analysis of the original FeP/Ni_2P hybrid catalyst. **a** Fe $2p^{3/2}$. **b** Ni $2p^{3/2}$. **c** O 1s.

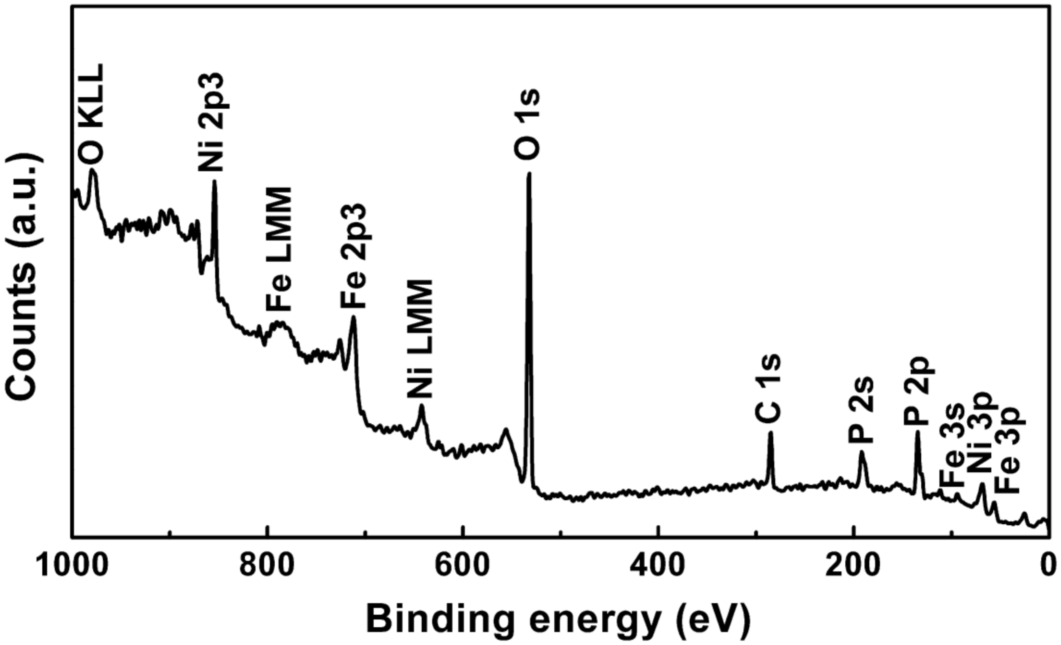

Supplementary Figure 4. XPS survey spectrum of the original FeP/Ni_2P hybrid catalyst.

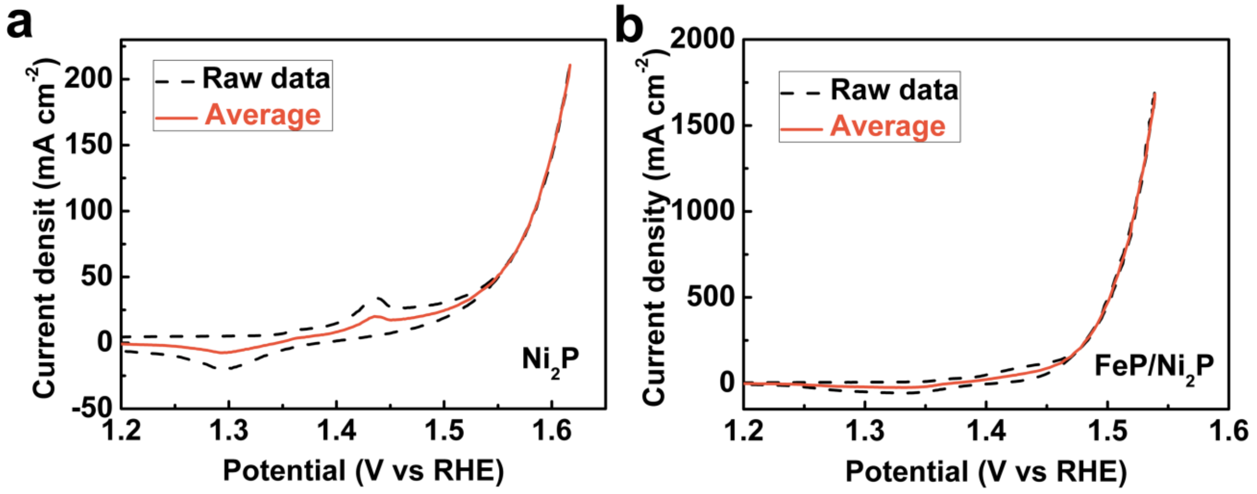

Supplementary Figure 5. A cyclic voltammetry (CV) curve (black) and corresponding average activity calculated from the CV curve (orange). **a** Ni_2P and **b** FeP/Ni_2P. Scan rate: 1 mV s^{-1}.

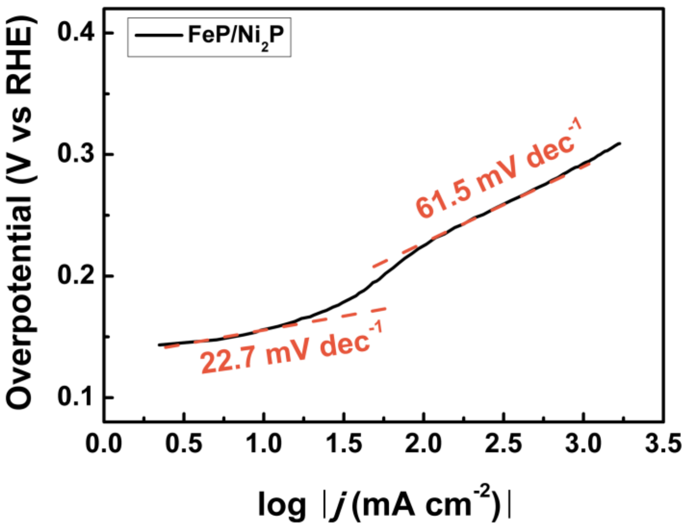

Supplementary Figure 6. Detailed Tafel slope analysis with a wide range of OER overpotentials.

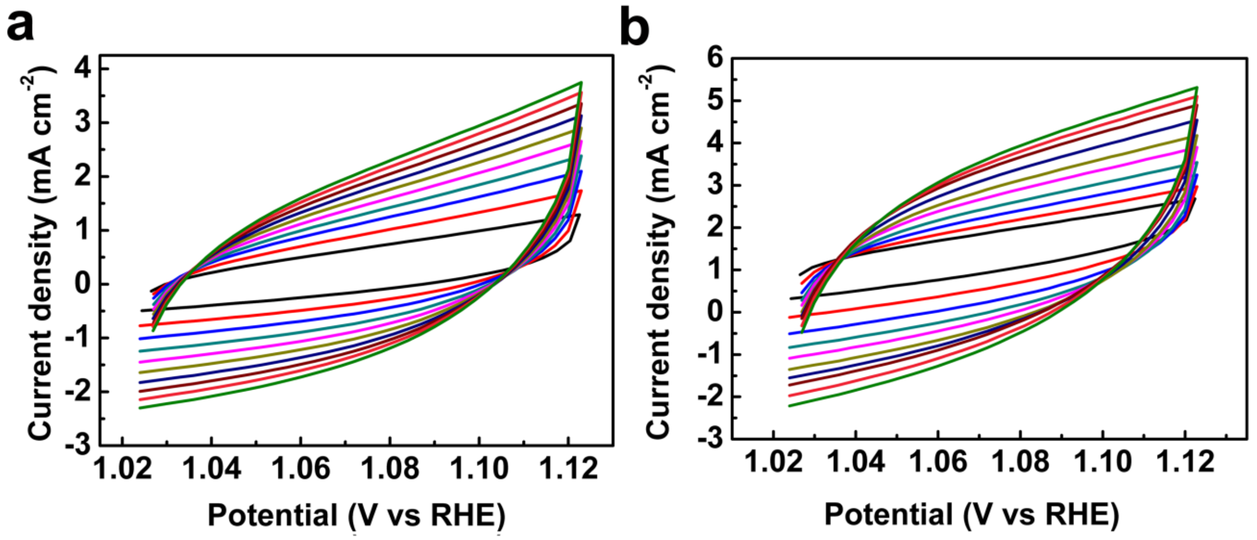

Supplementary Figure 7. Scan rate dependence of the current densities in the CV curves of different OER catalysts with scan rates ranging from 10 mV s^{-1} to 100 mV s^{-1} at intervals of 10 mV s^{-1}. **a** Ni_2P and **b** FeP/Ni_2P.

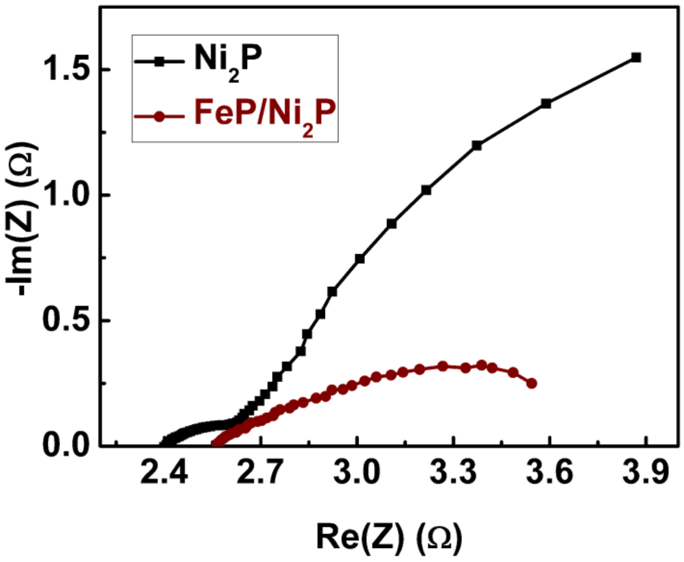

Supplementary Figure 8. Nyquist plots of electrochemical impedance spectroscopy (EIS) for OER catalyzed by Ni_2P and FeP/Ni_2P at an overpotential of 300 mV.

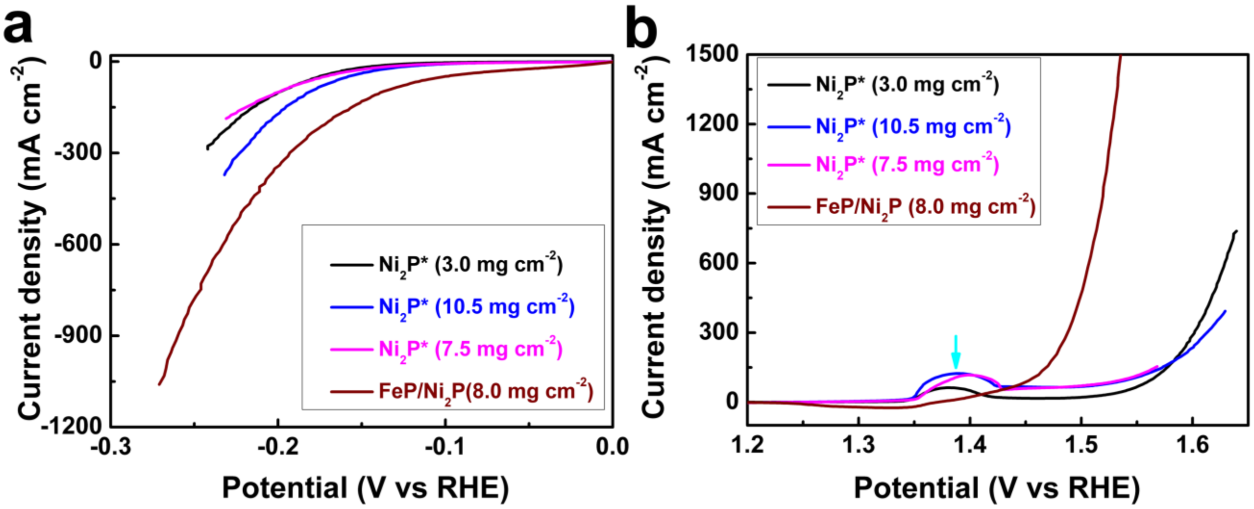

Supplementary Figure 9. The catalytic activities of Ni_2P^* catalysts grown on Ni foam with different loadings by using different $Ni(NO_3)_2$ precursor solutions. **a** HER polarization curves; **b** OER polarization curves.

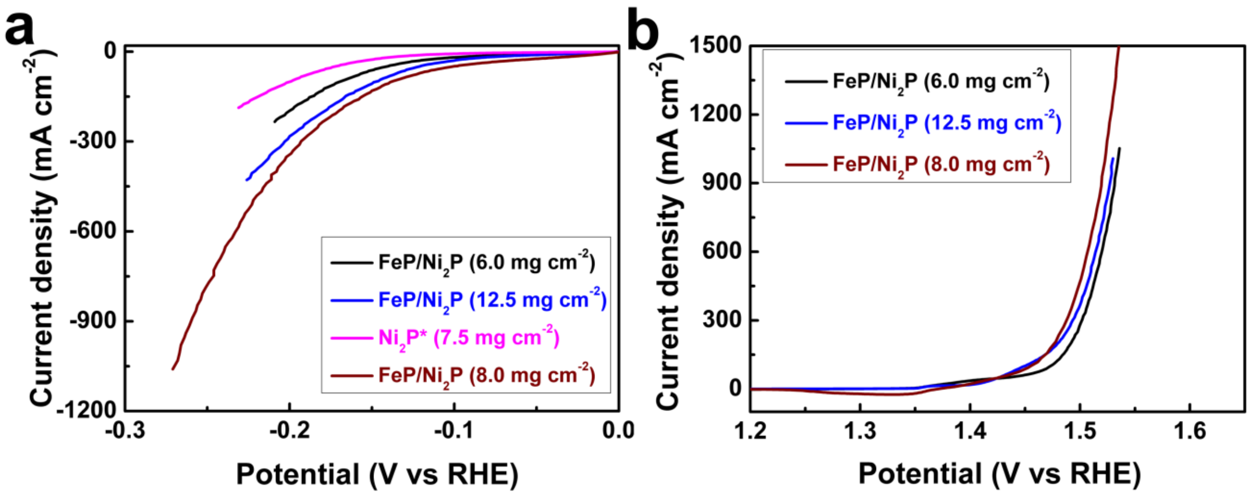

Supplementary Figure 10. The modulation on the catalytic performances of FeP/Ni_2P hybrids by tuning the loadings of FeP particles. It is found that 8 mg cm^{-2} is an optimal catalyst loading for FeP/Ni_2P hybrid grown on top of Ni foam. **a** HER polarization curves; **b** OER polarization curves.

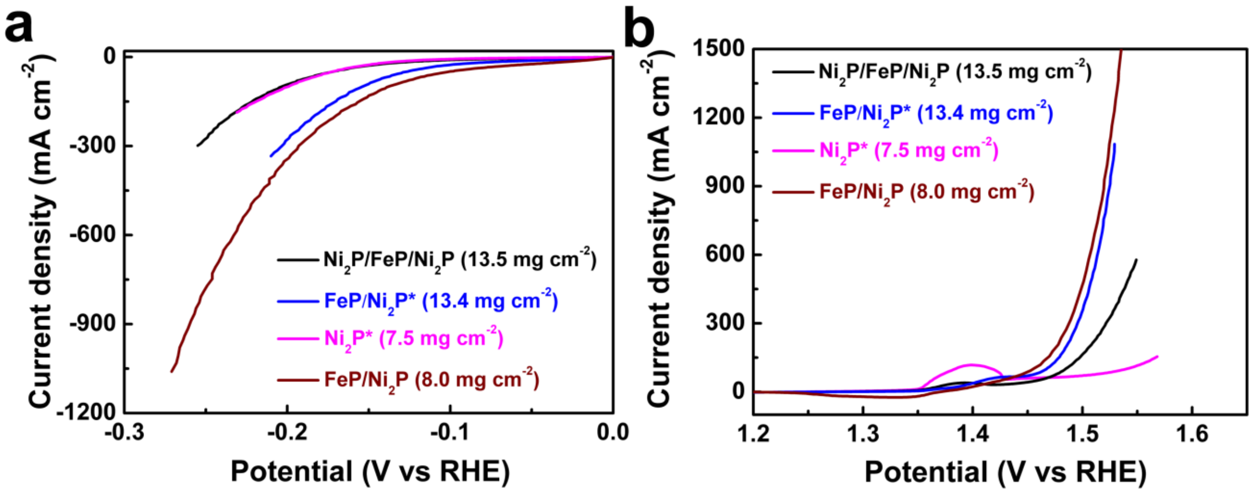

Supplementary Figure 11. The polarization curves of different catalysts showing the effects of Ni_2P and FeP order on the catalytic activities. **a** HER and **b** OER catalytic activities. Obviously, if Ni_2P was grown on top of FeP/Ni_2P (8.0 mg cm^{-2}), the as-obtained $Ni_2P/FeP/Ni_2P$ hybrid (13.5 mg cm^{-2}) shows much poorer catalytic activities compared to that when FeP was grown on Ni_2P^* (7.5 mg cm^{-2}) surface forming FeP/Ni_2P^* (13.4 mg cm^{-2}). The hybrid FeP/Ni_2P (8.0 mg cm^{-2}) is the sample that we investigated in our main texts.

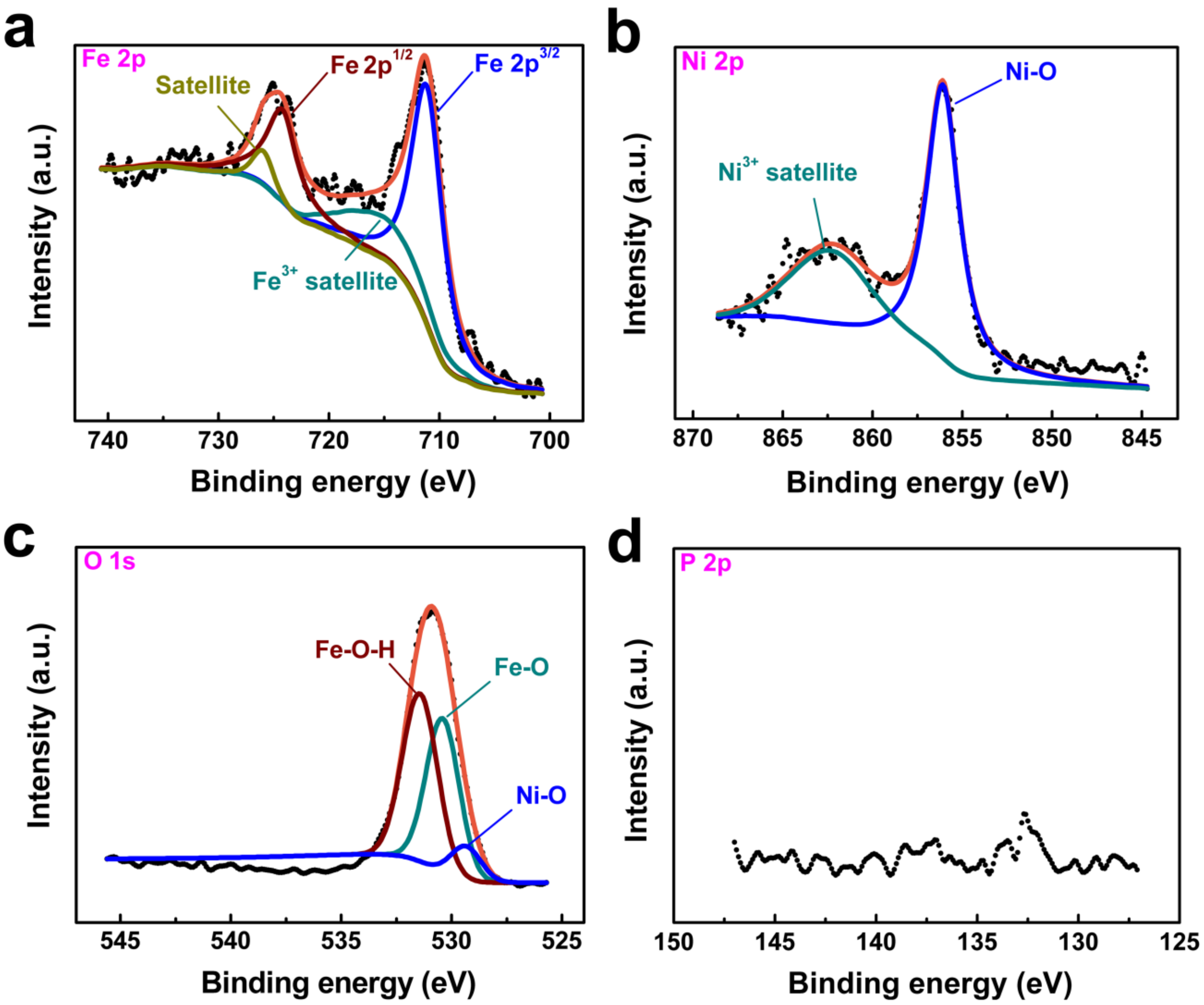

Supplementary Figure 12. XPS analysis of the post-OER samples. **a** Fe 2p region. **b** Ni $2p^{3/2}$ region. **c** O 1s region. **d** P 2p region. From these XPS spectra, we can deduce that the real active sites for the OER are possibly the mixed NiFe oxides/oxyhydroxides on the surface.

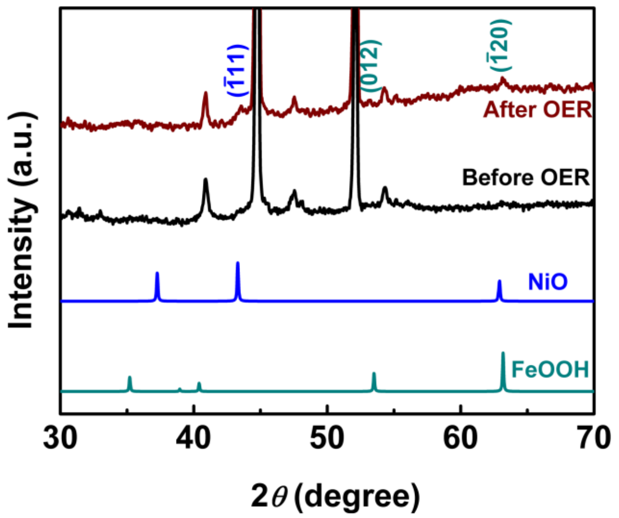

Supplementary Figure 13. XRD patterns of the FeP/Ni_2P nanoparticles after OER testing.

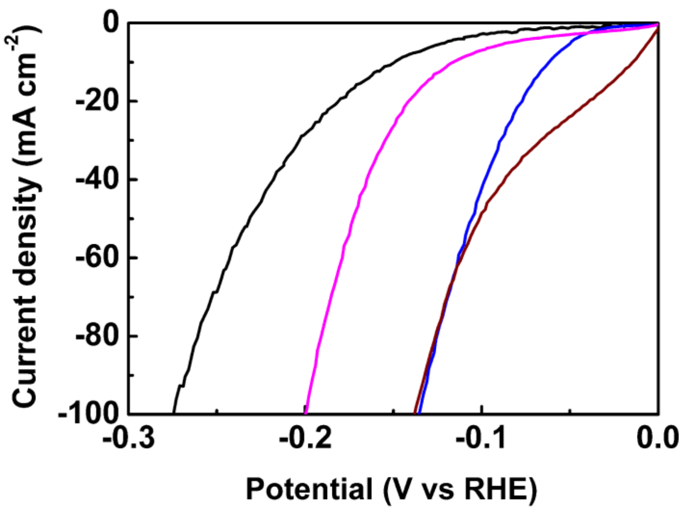

Supplementary Figure 14. Enlarged polarization curves of different HER electrocatalysts.

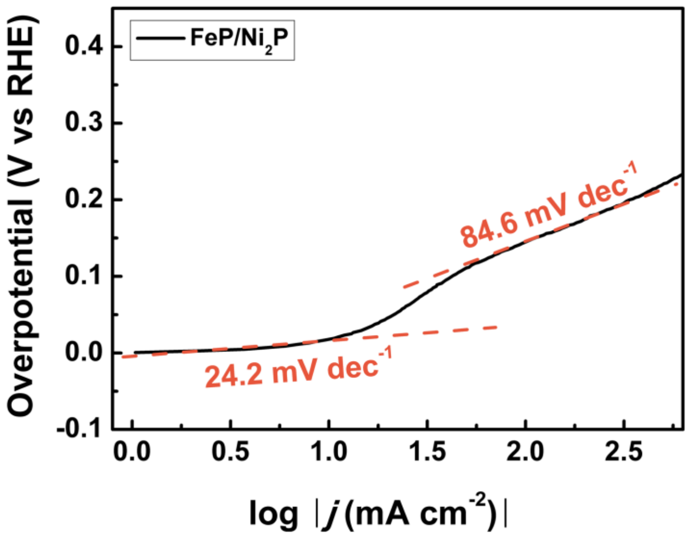

Supplementary Figure 15. Detailed Tafel slope analysis with a wide range of HER overpotentials.

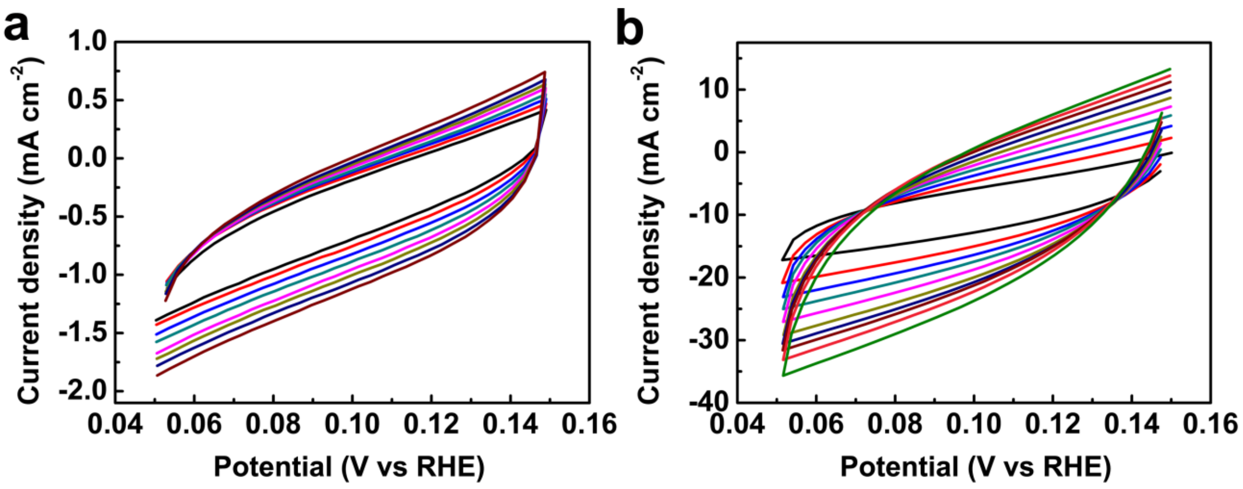

Supplementary Figure 16. Scan rate dependence of the current densities in the CV curves of different HER catalysts with scan rates ranging from 1 mV s^{-1} to 10 mV s^{-1} at intervals of 1 mV s^{-1}. **a** Ni_2P/Ni foam and **b** FeP/Ni_2P.

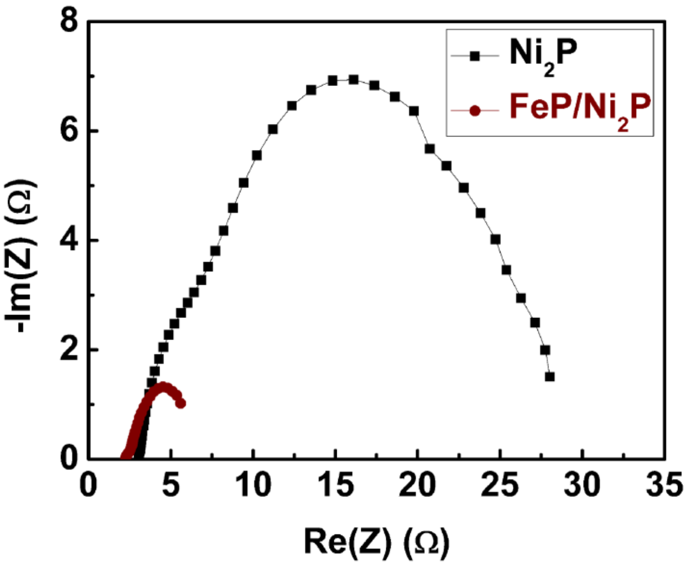

Supplementary Figure 17. Nyquist plots of Ni_2P and FeP/Ni_2P for HER measured at -150 mV vs RHE.

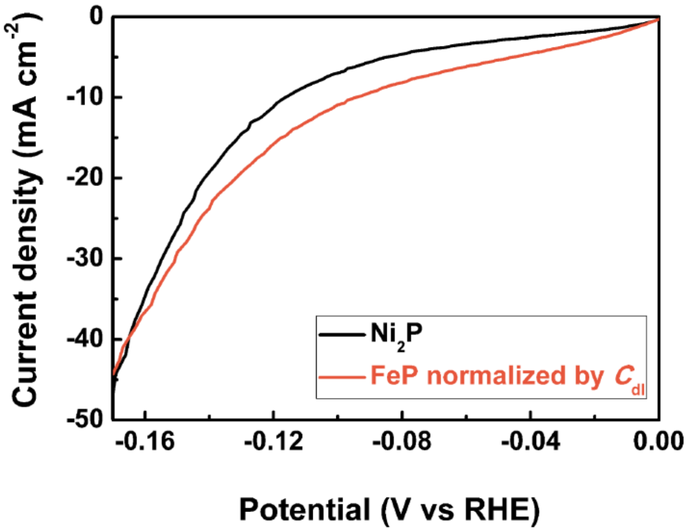

Supplementary Figure 18. Comparison of the catalytic HER activity with the same active surface area normalized by the double-layer capacitance (C_{dl}) difference between FeP/Ni_2P hybrid and just Ni_2P catalyst.

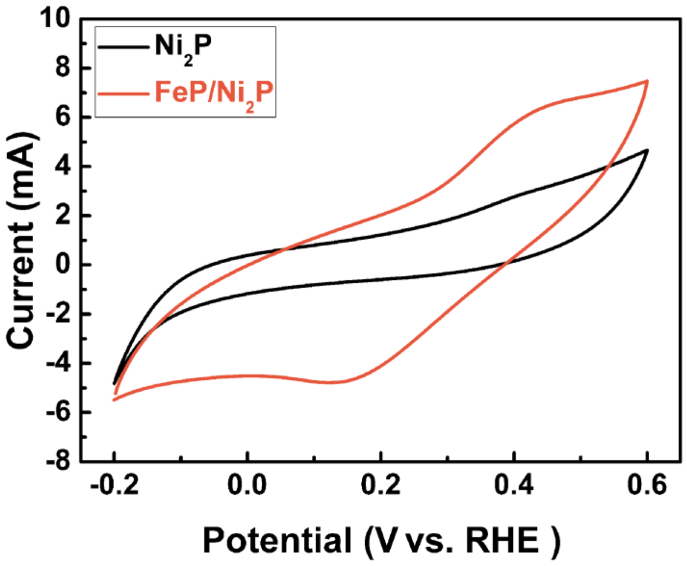

Supplementary Figure 19. CV curves recorded on the FeP/Ni_2P hybrid and pure Ni_2P electrodes in the potential ranges between -0.2 V vs RHE and 0.6 V vs RHE. The scan rate was 50 mV s^{-1}. Electrolyte: 1 M phosphate buffered saline (PBS).

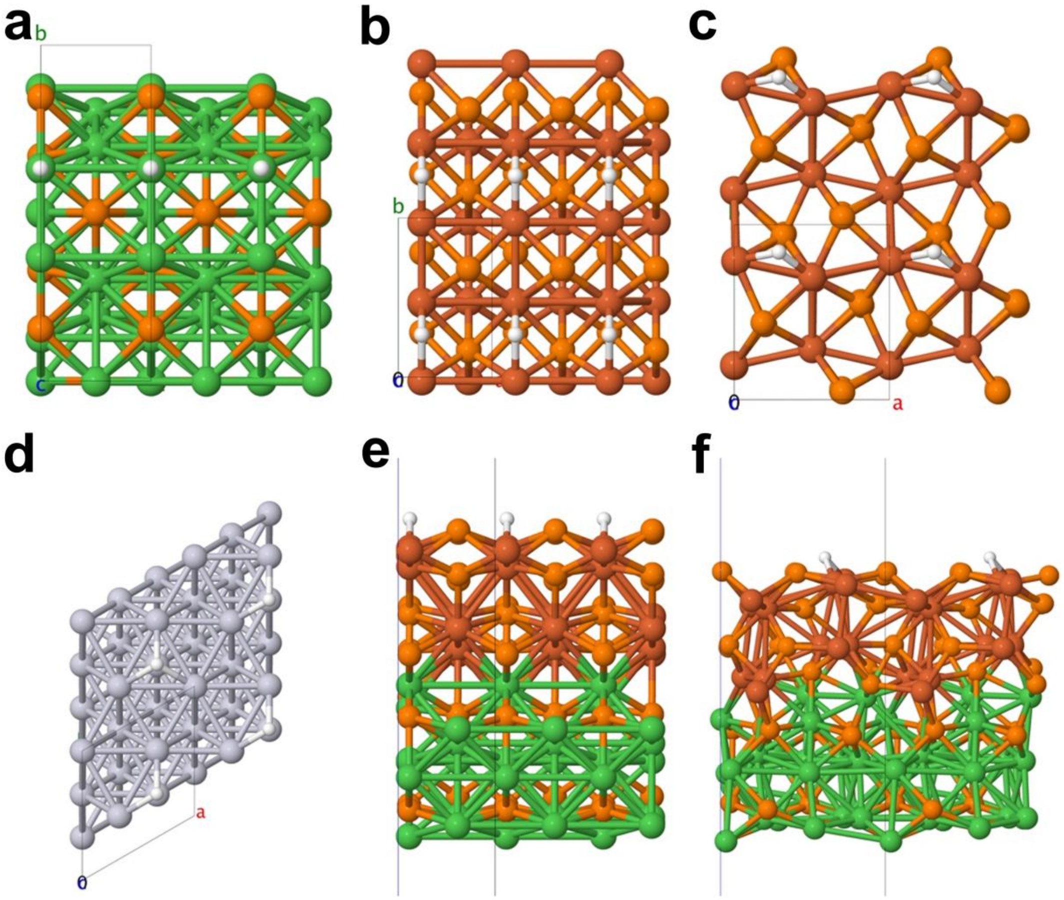

Supplementary Figure 20. Molecular structures of the systems calculated. **a** $Ni_2P(100)$; **b** FeP(001); **c** FeP(010); **d** Pt(111); **e** FeP(001) on $Ni_2P(100)$, side view; **f** FeP(010) on $Ni_2P(100)$, side view.

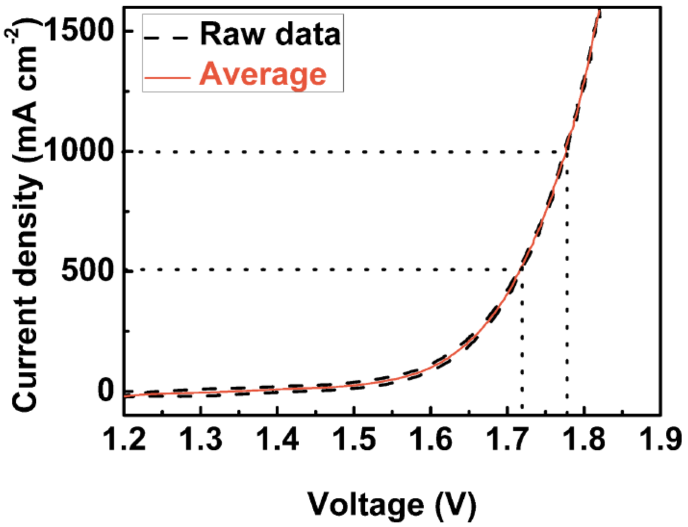

Supplementary Figure 21. A cyclic voltammetry (CV) curve (black) and corresponding average activity calculated from the CV curve (red) of FeP/Ni_2P as a bifunctional catalyst for overall water splitting. Scan rate: 1 mV s^{-1}.

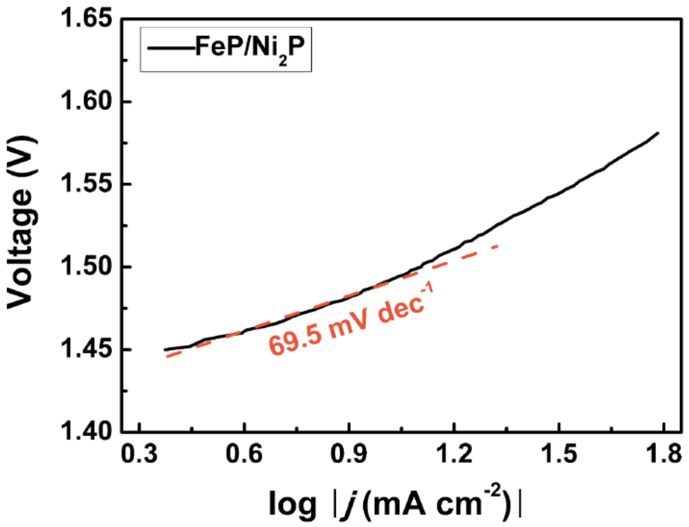

Supplementary Figure 22. Tafel analysis of the relevant polarization curve for overall water splitting.

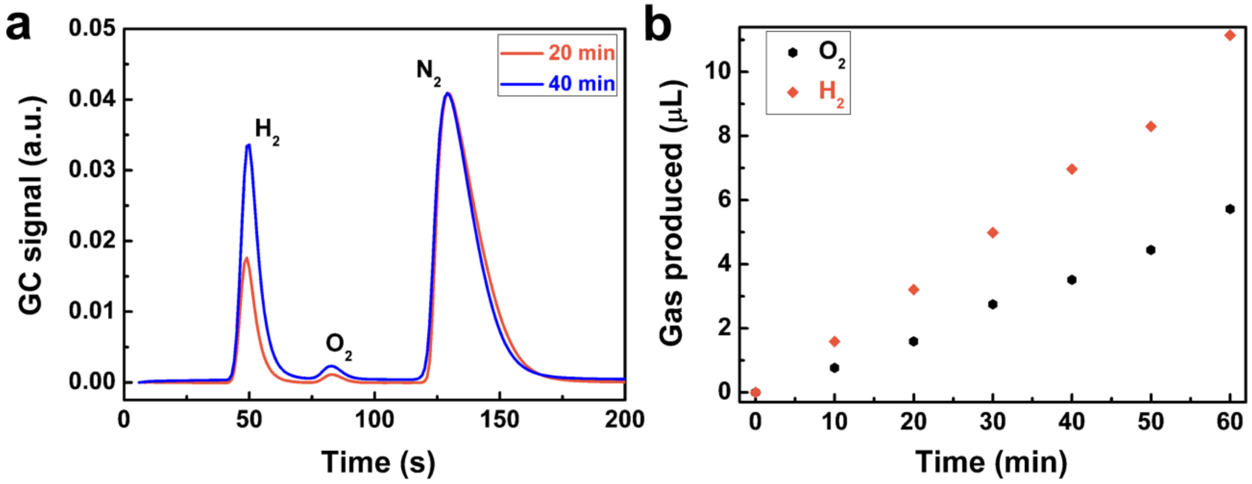

Supplementary Figure 23. Measurements of gas products from overall water splitting by gas chromatography (GC). **a** GC signals for the FeP/Ni_2P-based water alkaline electrolyzer after 20 and 40 min of overall water splitting. **b** The amounts of H_2 and O_2 gases versus time at a constant current density of 100 mA cm^{-2}. It is shown that there is a stoichiometric ratio around 2:1 between generated H_2 and O_2.

Supplementary Table 1. The distribution of surface oxidized species and metal phosphides quantified by the XPS survey spectrum, Fe $2p^{3/2}$ and Ni $3p^{3/2}$ XPS spectra of the original FeP/Ni_2P hybrid catalyst.

**Elements**	**Fe**		**Ni**		**P**	**O**	**C**
	**Fe-P**	**Fe-PO_x or oxide**	**Ni-P**	**Ni-PO_x**			
**Ratio**	**1.88**	**5.60**	**3.57**	**0.56**	**18.47**	**48.41**	**21.51**

Supplementary Table 2. Comparison of catalytic performance with the most recently reported OER catalysts. $\eta_{10,OER}$ corresponds to the overpotential of OER catalyzed at a current density of 10 mA cm^{-2}, while $j_{300,OER}$ corresponds to the current density at 300 mV overpotential for the OER.

OER catalysts	Electrolytes	$\eta_{10,OER}$ (mV)	Tafel (mV dec^{-1})	Source
FeP/Ni_2P	1.0 M KOH	154	22.7	Our work
Gelled FeCoW	1.0 M KOH	191	37	1
$Ni_xFe_{1-x}Se_2$-DO	1.0 M KOH	195	28	2
$NiCeO_x$–Au	1.0 M NaOH	270	-	3
Ni_2P nanoparticles	1.0 M KOH	290	59	4
Co_4N	1.0 M KOH	257	44	5
h-NiS_x	1.0 M KOH	180	96	6
FeP-rGO	1.0 M KOH	260	49.6	7
**Bifunctional catalysts for the OER**	**Electrolytes**	$\eta_{10,OER}$ **(mV)**	$j_{300,OER}$ **(mA cm^{-2})**	**Source**
FeP/Ni_2P	1.0 M KOH	154	1277	This work
Porous MoO_2	1.0 M KOH	260	41*	8
$Ni_{0.51}Fe_{0.49}P$ film	1.0 M KOH	239	80*	9
MoS_2/Ni_3S_2	1.0 M KOH	218	100*	10
CoP_2/rGO	1.0 M KOH	300	10	11
$NiCo_2S_4$ nanowire array	1.0 M KOH	260	19*	12
Electrodeposited CoP film	1.0 M KOH	345	0.5*	13
$NiCo_2O_4$	1.0 M KOH	290	24*	14
EG/$Co_{0.85}$Se/NiFe-LDH	1.0 M KOH	206	300*	15
NiFe LDH	1.0 M KOH	240	30*	16
NiFe LDH@DG10	1.0 M KOH	201	77.5*	17
NiFe LDH/Cu NW	1.0 M KOH	199	214*	18
$NiFeO_x$/CFP	1.0 M KOH	230	400*	19
NiP/Ni	1.0 M KOH	247	50*	20

Supplementary Table 3. Comparison of catalytic performance with the available non-noble HER catalysts in alkaline electrolytes. $\eta_{10,\ HER}$ corresponds to the overpotential of HER catalyzed at 10 mA cm^{-2}, and $j_{200,\ HER}$ is related to the current density at 200 mV overpotential.

HER catalysts	Electrolytes	$\eta_{10,\ HER}$ (mV)	Tafel (mV dec^{-1})	Source
FeP/Ni_2P	1.0 M KOH	14	24.2	This work
$NiCo_2P_x$ Nanowires	1.0 M KOH	58	34.3	21
$Ni_{1-x}Co_xSe_2$ nanosheet	1.0 M KOH	85	52.0	22
CoP nanowire/CC	1.0 M KOH	209	129.0	23
Co/CoP nanocrystals	1.0 M KOH	135	64.0	24
FeP nanowire arrays	1.0 M KOH	194	75	25
$MoNi_4/MoO_2$ cuboids	1.0 M KOH	15	30.0	26
MoP crystals	1.0 M KOH	~ 140	48.0	27
Ni_5P_4 (pellet)	1.0 M KOH	49	98.0	28
Nanoporous Co_2P	1.0 M KOH	60	40.0	29
**Bifunctional catalysts for the HER**	**Electrolytes**	$\eta_{10,HER}$ **(mV)**	$j_{200,HER}$ **(mA cm^{-2})**	**Source**
FeP/Ni_2P	1.0 M KOH	14	346*	This work
Porous MoO_2	1.0 M KOH	27	132*	8
$Ni_{0.51}Fe_{0.49}P$ film	1.0 M KOH	82	236*	9
MoS_2/Ni_3S_2	1.0 M KOH	110	92*	10
CoP_2/rGO	1.0 M KOH	88	84*	11
$NiCo_2S_4$ nanowire array	1.0 M KOH	210	7.4*	12
Electrodeposited CoP film	1.0 M KOH	94	480*	13
$NiCo_2O_4$	1.0 M KOH	110	52*	14
EG/$Co_{0.85}$Se/NiFe-LDH	1.0 M KOH	260	4.3*	15
NiFe LDH	1.0 M KOH	210	8.2*	16
NiFe LDH@DG10	1.0 M KOH	66	60*	17
NiFe LDH/Cu NW	1.0 M KOH	116	124*	18
$NiFeO_x$/CFP	1.0 M KOH	88	62*	19
NiP/Ni	1.0 M KOH	130	134*	20

Supplementary Table 4. Calculated ΔG_H in eV.

**ΔG_H (eV)**	**Acidic condition (pH=0)**	**Basic condition (pH=14)**
Ni_2P(100)	0.399	0.306
FeP(001)	-0.059	-0.057
FeP(010)	-0.221	-0.237
FeP(001)/Ni_2P	-0.279	-0.255
FeP(010)/Ni_2P	-0.238	-0.230
Pt	-0.184	-0.135

Supplementary Table 5. Comparison of the HER, OER and overall water splitting activities with available robust bifunctional catalysts. $\eta_{10,HER}$, $\eta_{10,OER}$, $\eta_{10,overall}$, $\eta_{100,overall}$ and $j_{1.7,overall}$ correspond to the overpotentials of HER, OER catalyzed at 10 mA cm^{-2}, the cell voltages at 10 and 100 mA cm^{-2}, and current density at 1.7 V for the overall water splitting, respectively.

Catalyst	Electrolytes	$\eta_{10,\ HER}$ (mV)	$\eta_{10,\ OER}$ (mV)	$\eta_{10,\ overall}$ (V)	$\eta_{100,\ overall}$ (V)	$j_{1.7,\ overall}$ (mA cm^{-2})	Source
FeP/Ni_2P	1.0 M KOH	14	154	1.42	1.602	406	This work
Porous MoO_2	1.0 M KOH	27	260	1.53	1.8*	67*	8
$Ni_{0.51}Fe_{0.49}P$ film	1.0 M KOH	82	239	1.57	1.71*	87*	9
MoS_2/Ni_3S_2	1.0 M KOH	110	218	1.56	1.71*	91.4*	10
CoP_2/rGO	1.0 M KOH	88	300	1.56	1.912*	31*	11
$NiCo_2S_4$ nanowire array	1.0 M KOH	210	260	1.63	2.097*	16*	12
Electrodeposited Co-P film	1.0 M KOH	94	345	1.64*	1.745*	42*	13
$NiCo_2O_4$	1.0 M NaOH	110	290	1.65	1.842*	16*	14
EG/Co0.85Se/NiFe-LDH	1.0 M KOH	260	206	1.67	1.907*	16.6*	15
NiFe LDH	1.0 M NaOH	210	240	1.7	2.241*	10	16
NiFe LDH@DG10	1.0 M KOH	66	201	1.44*	1.87*	60*	17
NiFe LDH/Cu NW	1.0 M KOH	116	199	1.54	1.69*	111*	18
$NiFeO_x$/CFP	1.0 M KOH	88	230	1.51	1.73*	70*	19
NiP/Ni	1.0 M KOH	130	247	1.61	2.102*	24*	20

*The data were calculated according to the curves given in the literature.

Supplementary Note 1: Calculation of turn over frequency (TOF).

Supposing that every active site was accessible to the electrolyte, the TOF values can be calculated by the following formula:

$$TOF = \frac{1}{2}\frac{I}{nF} \qquad (1)$$

where these physical variables *F*, *n*, and *I* are corresponding to the Faraday constant (~ 96485 C/mol), the number of active sites (mol), and the current (A) during the LSV measurement in 1 M KOH, respectively. The factor 1/2 is due to fact that two electrons are required to form one hydrogen molecule from two protons.

The number of active sites was determined by an electrochemical method.[30,31] The CV curves were measured in 1M PBS electrolyte (pH = 7). Due to the difficulty in assigning the observed peaks to a given redox couple, the number of active sites is nearly proportional to the integrated voltammetric charges (cathodic and anodic) over the CV curves. Supposing a one-electron process for both reduction and oxidation, we can get the upper limit of the number of active sites (n) based on the follow equation:

$$n = \frac{Q}{2F} \qquad (2)$$

where *F* and *Q* are the Faraday constant and the whole charge of CV curve, respectively. By this equation and the CV curves, we can obtain the number of active sites for the FeP/Ni_2P hybrid is around 3.71×10^{-7} mol, while this value is changed to 1.47×10^{-7} mol for the pure Ni_2P catalyst, meaning that the FeP/Ni_2P hybrid has active sites 2.5 times that of just Ni_2P catalyst.

Supplementary references

1. Zhang, B. *et al.* Homogeneously dispersed multimetal oxygen-evolving catalysts. *Science* **352**, 333-337 (2016).
2. Xu, X., Song, F. & Hu, X. L. A nickel iron diselenide-derived efficient oxygen-evolution catalyst. *Nat. Commun.* **7**, 12324 (2016).
3. Ng, J. W. D. *et al.* Gold-supported cerium-doped NiO_x catalysts for water oxidation. *Nature Energy* **1**, 16053 (2016).
4. Stern, L. A., Feng, L. G., Song, F. & Hu, X. L. Ni_2P as a Janus catalyst for water splitting: the oxygen evolution activity of Ni_2P nanoparticles. *Energy Environ. Sci.* **8**, 2347-2351 (2015).
5. Chen, P. Z. *et al*. Metallic Co_4N porous nanowire arrays activated by surface oxidation as electrocatalysts for the oxygen evolution reaction. *Angew. Chem. Int. Ed.* **54**, 14710-14714 (2015).
6. You, B. & Sun, Y. J. Hierarchically porous nickel sulfide multifunctional superstructures. *Adv. Energy Mater*. **6**, 1502333 (2016).
7. Masud, J., Umapathi, S., Ashokaan, N. & Nath, M. Iron phosphide nanoparticles as an efficient electrocatalyst for the OER in alkaline solution. *J. Mater. Chem. A* 4, 9750-9754 (2016).
8. Jin, Y. S. *et al*. Porous MoO_2 nanosheets as non-noble bifunctional electrocatalysts for overall Water Splitting. *Adv. Mater*. **28**, 3785-3790 (2016).
9. Yu, J. et al. Ternary metal phosphide with triple-layered structure as a low-cost and efficient electrocatalyst for bifunctional water splitting. *Adv. Funct. Mater.* **26**, 7644-7651 (2016).
10. Zhang, J. *et al*. Interface engineering of MoS_2/Ni_3S_2 heterostructures for highly enhanced electrochemical overall-water-splitting activity. *Angew*. *Chem*. *Int*. *Ed*. **55**, 6702-6707 (2016).

11. Wang, J. M., Yang, W. R. & Liu, J. Q. CoP_2 nanoparticles on reduced graphene oxide sheets as a super-efficient bifunctional electrocatalyst for full water splitting. *J. Mater. Chem. A* **4**, 4686-4690 (2016).

12. Sivanantham, A., Ganesan, P. & Shanmugam, S. Hierarchical $NiCo_2S_4$ nanowire arrays supported on Ni foam: an efficient and durable bifunctional electrocatalyst for oxygen and hydrogen evolution reactions. *Adv. Funct. Mater.* **26**, 4661-4672 (2016).

13. Jiang, N., You, B., Sheng, M. L. & Sun, Y. J. Electrodeposited cobalt-phosphorous-derived films as competent bifunctional catalysts for overall water splitting. *Angew. Chem. Int. Ed.* **54**, 6251-6254 (2015).

14. Gao, X. H. *et al*. Hierarchical $NiCo_2O_4$ hollow microcuboids as bifunctional electrocatalysts for overall water splitting. *Angew. Chem. Int. Ed.* **55**, 6290-6294 (2016).

15. Hou, Y. et al. Vertically oriented cobalt selenide/NiFe layered-double-hydroxide nanosheets supported on exfoliated graphene foil: an efficient 3D electrode for overall water splitting. *Energy Environ. Sci.* **9**, 478-483 (2016).

16. Luo, J. S. *et al*. Water photolysis at 12.3% efficiency via perovskite photovoltaics and Earth-abundant catalysts. *Science* **345**, 1593-1596 (2014).

17. Jia, Y. *et al*. A heterostructure coupling of exfoliated Ni-Fe hydroxide nanosheet and defective graphene as a bifunctional electrocatalyst for overall water splitting. *Adv. Mater.* **29**, 1700017 (2017).

18. Yu, L. *et al*. Cu nanowires shelled with NiFe layered double hydroxide nanosheets as bifunctional electrocatalysts for overall water splitting. *Energy Environ. Sci.* **10**, 1820-1827 (2017).

19. Wang, H. T. *et al.,* Bifunctional non-noble metal oxide nanoparticle electrocatalysts through lithium-induced conversion for overall water splitting. *Nat. Commun.* **6**, 7261 (2015).

20. Chen, G. F. et al. Efficient and stable bifunctional electrocatalysts Ni/Ni_xM_y (M = P, S) for overall water splitting. *Adv. Funct. Mater.* **26**, 3314-3323 (2016).

21. Zhang, R. *et al*. Ternary $NiCo_2P_x$ Nanowires as pH-Universal Electrocatalysts for Highly Efficient Hydrogen Evolution Reaction. *Adv. Mater.* **29**, 1605502 (2017).

22. Liu, B. *et al*. Nickel-cobalt diselenide 3D mesoporous nanosheet networks supported on Ni foam: an all-pH highly efficient integrated electrocatalyst for hydrogen evolution. *Adv. Mater.* **29**, 1606521 (2017).

23. Tian, J. Q., Liu, Q., Asiri, A. M. & Sun, X. P. Self-supported nanoporous cobalt phosphide nanowire arrays: an efficient 3D hydrogen-evolving cathode over the wide range of pH 0-14. *J. Am. Chem. Soc.* **136**, 7587-7590 (2014).

24. Wang, H. *et al.* Nitrogen-doped nanoporous carbon membranes with Co/CoP Janus-type nanocrystals as hydrogen evolution electrode in both acidic and alkaline environments. *ACS Nano* **11**, 4358-4364 (2017).

25. Son, C. Y., Kwak, I. H., Lim, Y. R. & Park, J. FeP and FeP_2 nanowires for efficient electrocatalytic hydrogen evolution reaction. *Chem. Commun.* **5**2, 2819-2822 (2016).

26. Zhang, J. *et al.* Efficient hydrogen production on $MoNi_4$ electrocatalysts with fast water dissociation kinetics. *Nat. Commun.* **8**, 15437 (2017).

27. Xiao, P. *et al*. Molybdenum phosphide as an efficient electrocatalyst for the hydrogen evolution reaction. *Energy Environ. Sci.* **7**, 2624-2629 (2014).

28. Laursen, A. B. *et al.* Nanocrystalline Ni_5P_4: a hydrogen evolution electrocatalyst of exceptional efficiency in both alkaline and acidic media. *Energy Environ. Sci.* **8**, 1027-1034

(2015).

29. Tan, Y. W. *et al*. 3D nanoporous metal phosphides toward high-efficiency electrochemical hydrogen production. *Adv. Mater.* **28**, 2951-2955 (2016).

30. Merki, D., Fierro, S., Vrubel, H. & Hu, X. L. Amorphous molybdenum sulfide films as catalysts for electrochemical hydrogen production in water. *Chem. Sci.* **2**, 1262-1267 (2011).

31. Chen, Y. Y. et al. Self-templated fabrication of $MoNi_4/MoO_{3-x}$ nanorod arrays with dual active components for highly efficient hydrogen evolution. *Adv. Mater.* **29**, 1703311 (2017).
